# An Ab Initio Study of Aqueous Copper(I) Speciation in the Presence of Chloride

**DOI:** 10.3390/molecules30153147

**Published:** 2025-07-27

**Authors:** Daniel C. M. Whynot, Christopher R. Corbeil, Darren J. W. Mercer, Cory C. Pye

**Affiliations:** Department of Chemistry, Saint Mary’s University, 923 Robie Street, Halifax, NS B3H 3C3, Canada

**Keywords:** ab initio, density functional, copper(I) chloro complexes, structure, vibrational frequencies

## Abstract

The determination of multiple energy minima on complex potential energy surfaces is challenging. A systematic desymmetrization procedure was employed to find stationary points on the copper(I) + chloride + water potential energy surface using HF, MP2, and B3LYP methods in conjunction with the 6-31G*, 6-31+G*, and 6-311+G* basis sets. Comparison with experimental results demonstrated that the speciation of copper(I) in the presence of chloride and water may be formulated as [CuCl(H_2_O)]^0^, [CuCl_2_]^−^, and [CuCl_3_]^2−^. Our results indicate that the combination of the MP2 method along with basis sets containing diffuse functions gives excellent agreement with experimental Cu-Cl distances and vibrational frequencies. Poorer results were obtained at the HF levels and/or using the 6-31G* basis set.

## 1. Introduction

Speciation in chemistry refers to the distribution of an element amongst defined chemical species in a system in some medium. In water, this is called aqueous speciation. For example, solid sodium chloride, NaCl(s), will dissolve in water to give NaCl(aq). Above a certain concentration, however, not all of the sodium chloride will dissolve, and some solid will remain as NaCl(s) above the freezing point of pure water. At lower temperatures, the solid can exist as the dihydrate NaCl•2H_2_O(s) [1]. In this structure, each sodium ion is surrounded by four water molecules and two chloride ions. Recently, other hydrates of sodium chloride, potentially relevant to Jupiter’s moons, were identified at low temperatures and high pressures: NaCl•8.5H_2_O(s) and NaCl•13H_2_O(s) [2]. In both phases, the sodium exists as a hexaaqua ion, [Na(H_2_O)_6_]^+^. Suppose we restrict ourselves to the aqueous phase. In that case, it is known that NaCl(aq) at near-ambient conditions can be described as an equimolar mixture of Na^+^(aq) and Cl^−^(aq) ions. It is believed that the sodium ion can also be described as a hexaaqua ion, with the water molecules forming a hydration shell around the sodium ion. In both the crystal and aqueous solution, the environment around the aqua ion may influence the bond distances and vibrational frequencies [3].

An alternate way to study speciation for ionic compounds is to fix the amount of cation and vary the amount of anion. The speciation of copper(I) in aqueous solution in the presence of excess chloride ion was recently investigated by some of the authors [4]. The Cu^+^(aq) ion is difficult to study experimentally, as it tends to disproportionate into Cu^2+^(aq) and Cu^0^(s) in aqueous solution and can be easily oxidized by adventitious oxygen. However, the presence of halide ions stabilizes the +1 oxidation state of copper. Therein, it was shown using a combination of Raman spectroscopy and ab initio calculations that the two bands observed at 297 cm^−1^ and 247 cm^−1^ were the totally symmetric Cu-Cl stretching motions of the linear D_∞h_ CuCl_2_^−^(aq) and trigonal planar D_3h_ CuCl_3_^2−^(aq) species, respectively. A less intense band at 350 cm^−1^ was assigned to the neutral CuCl(H_2_O)^0^(aq) species. A systematic desymmetrization procedure was developed and applied by the authors to the possible aquacopper(I) species Cu(H_2_O)*_n_*^+^, *n* = 1–6 [5]. Therein, it was shown that the most likely structure should be the diaquacopper(I) ion, Cu(H_2_O)_2_^+^.

Desymmetrization, as first developed in Ref. [5], uses group theory to systematically lower the symmetry of a high symmetry structure, which is a stationary point on the potential energy surface, to another lower-energy stationary point. A stationary point is a structure in which the potential energy gradient vector is zero. Stationary points may be characterized by the number of negative eigenvalues of the energy second-derivative matrix with respect to the nuclear motions. If all eigenvalues are positive, then the structure is a minimum on the potential energy surface. If one eigenvalue is negative, then the structure is called a transition structure. If *N* eigenvalues are negative, the structure is called an *N*-th order saddle point. The vibrational frequencies of a stationary point, being related to these eigenvalues, are typically used to characterize the stationary point because they can be related to infrared and Raman spectra. Locally stable structures have all positive vibrational frequencies, whereas a transition state would have one imaginary vibrational frequency corresponding to the maximum along the reaction coordinate (corresponding eigenvector). Distortion along this reaction coordinate from the maximum would result in a lowering of the energy, which sometimes coincides with a lowering of symmetry. Lowering the symmetry (or descent in symmetry) is the reduction in the order of the point group symmetry. For example, the C_2v_ point group has order 4 (there are 4 symmetry operations), whereas its subgroups C_s_ and C_2_ are both of order 2. An illustrative example well-known to students of organic chemistry via Newman projections would be the eclipsed form of ethane, which has D_3h_ symmetry (order 12). This structure has an imaginary frequency corresponding to the internal rotation of the irreducible representation A_1_”. In this form, the dihedral angle H-C-C-H is 0°. A slight distortion along this vibrational mode results in a D_3_ structure (order 6) and a lowering of energy. Once this angle reaches 60°, a potential energy minimum is reached, called the staggered form, and we have an ascent in symmetry to D_3d_ (order 12). For structures with multiple imaginary frequencies, the advantages of a systematic procedure for desymmetrization become evident.

In this paper, we will further explore chlorocopper(I) complexes, examining the effect of the waters of solvation on the coordination chemistry of chlorocopper complexes, in particular, the Cu-Cl distances and vibrational frequencies, using the techniques developed in Ref. [5].

## 2. Results

In the text, tables, and figures below, the structures are described by stoichiometry and charge, point group symmetry and numerical label (if more than one exists) corresponding to the stoichiometry, and in cases where not all ligands are bound, the coordination pattern [*m* + *n*], where *m* refers to the number of ligands directly coordinated to the copper(I) ion and *n* refers to those not directly coordinated to the copper(I) ion but interacting with the ligands. In some cases, *n* may be appended by Cl if a chloride ligand dissociates.

### 2.1. Structures of Monochloroaquacopper(I) Complexes, CuCl(H_2_O)_n_, n = 1–5, 7

**Figure 1:**
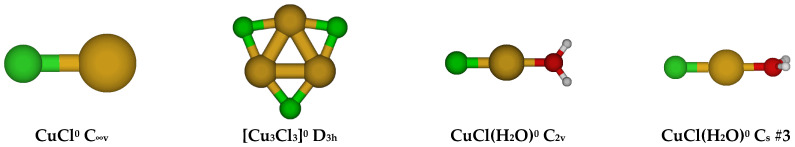
Chlorocopper(I) structures, CuCl(H_2_O)*_n_*^0^, *n* = 0, 1, and Cu_3_Cl_3_ (to be discussed later). In this and later figures, a bold label indicates a local minimum of potential energy at some level of theory. The atom color legend is as follows: copper is yellow, chlorine is green, oxygen is red, and hydrogen is white.

The simplest chlorocopper(I) complex, CuCl^0^, is of C_∞v_ symmetry (see Figure 1). When a water molecule is added to the copper ion opposite to the chloride to give CuCl(H_2_O)^0^, the symmetry reduces to C_2v_. This structure is usually unstable (except at HF/6-311+G*) and possesses an imaginary B_1_ mode. Desymmetrization along this mode gives the stable C_s_ #3 structure, in which a slight pyramidalization of the oxygen atom has occurred. Excited configuration structures corresponding to high-energy C_s_ #1 and #2 were also found in which the Cl-Cu-O angle was approximately 90 degrees.

**Figure 2:**
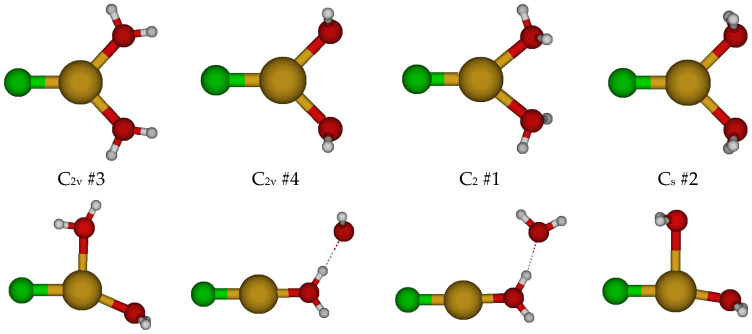

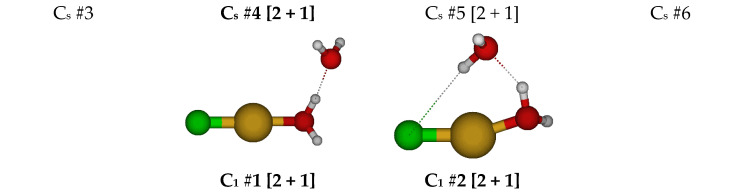
Chlorocopper(I) structures, CuCl(H_2_O)*_n_*^0^, *n* = 2. Several excited configuration structures were also found.

For CuCl(H_2_O)_2_^0^, we first considered two different C_2v_ structures in which the water molecules are either in the ClCuO_2_ plane or are bisected by it (Figure 2). These are close in energy but usually possess imaginary A_2_, B_1_, and B_2_ modes, suggesting desymmetrization to either a C_2_ or one of two C_s_ structures (#2, #3). The C_2_ #1 structure has an imaginary B mode at all levels of theory. The C_s_ #2 structure (close in structure to C_2v_ #3) has two imaginary A” frequencies. The C_s_ #3 structure only exists at a couple of levels of theory and dissociates a water molecule to become C_s_ #4 [2 + 1]. Most of these C_s_ structures contain an imaginary mode and become a [2 + 1] C_1_ #1 or #2 structure upon desymmetrization. These differ in that the ClCuO angle bends so that one of the hydrogen atoms of the water may interact with the CuCl part of the molecule at some levels of theory. These results suggest that CuCl(aq) is dicoordinate [CuCl(H_2_O)]^0^ in aqueous solution and that attempts to place a second water molecule in the first coordination shell of copper result in migration to the second coordination shell.

**Figure 3:**
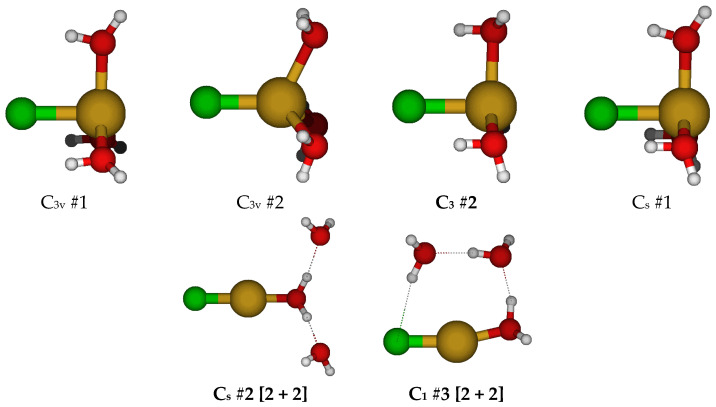
Chlorocopper(I) structures, CuCl(H_2_O)*_n_*^0^, *n* = 3.

For CuCl(H_2_O)_3_^0^, we first considered two different C_3v_ structures in which the water molecules are either in the three σ_v_ planes or are bisected by them (Figure 3). Both of these structures have one imaginary A_2_ mode and at least one imaginary E mode. Along the A_2_ mode, they desymmetrize to the common C_3_ #2 structure. This structure is a local minimum at some levels of theory (HF; B3LYP/6-311+G*) but contains an imaginary E mode at others (MP2; B3LYP/6-31G* and 6-31+G*). Desymmetrization of C_3v_ #1 and #2 along the E mode would give one of two C_s_ structures (#1 and #2, respectively). At some levels of theory, C_s_ #1 becomes [3 + 1], whereas C_s_ #2 becomes [2 + 2] at nearly all levels of theory except HF/6-31G* and B3LYP/6-31+G*. The C_3_ structure can desymmetrize along the E mode to give a C_1_ #3 [2 + 2] structure, which exists at all levels of theory.

**Figure 4:**
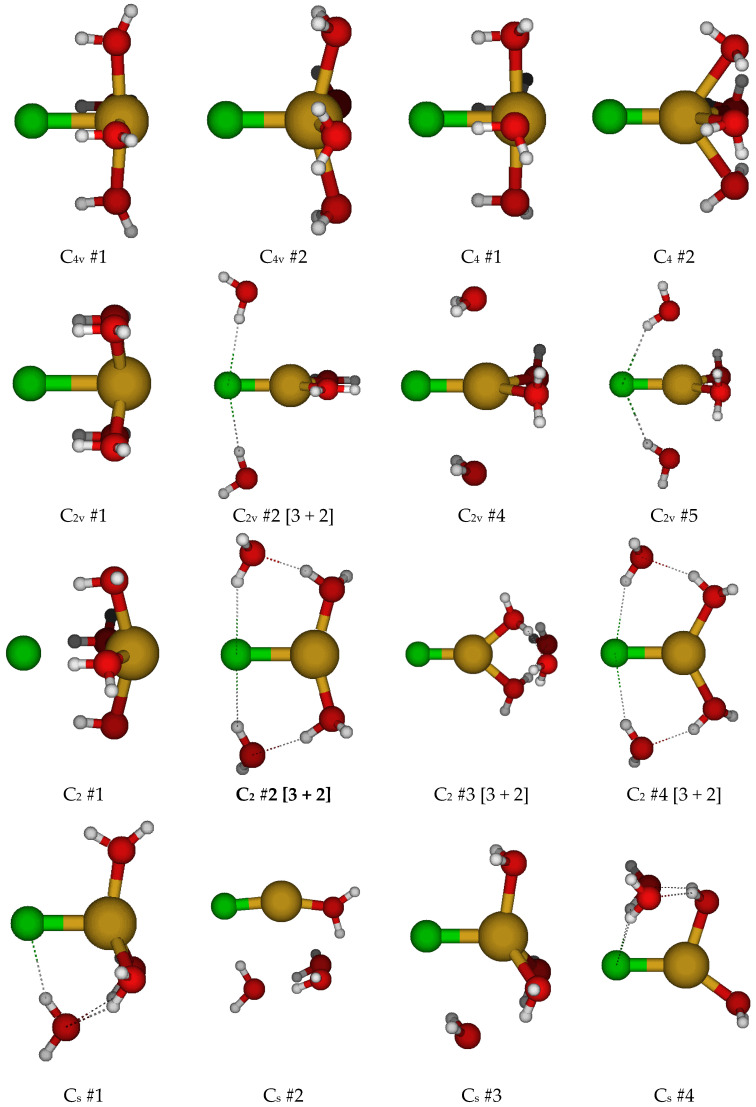

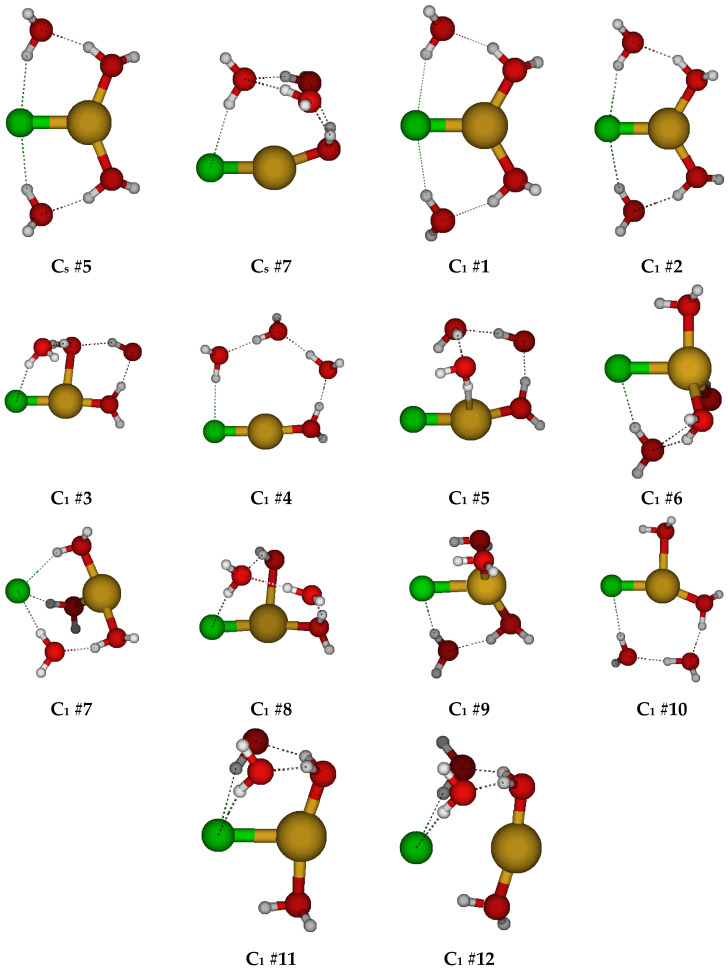
Chlorocopper(I) structures, CuCl(H_2_O)*_n_*^0^, *n* = 4.

For CuCl(H_2_O)_4_^0^, we first considered two different C_4v_ structures in which the water molecules are either in the three σ_v_ planes or are bisected by them (Figure 4). Both of these had imaginary A_2_, E, and B_1_ (and sometimes B_2_) modes. Along the A_2_ mode, these desymmetrize to form one of two C_4_ structures, #1 and #2, respectively, with some coalescence between them. They would desymmetrize along the B_1_ and B_2_ modes to form C_2v_ structures #1–#4, and along the E mode, the C_2v_ #5 structures (via C_s_). For C_4_ #2, the waters form a cyclic water tetramer. The C_4_ #1 structure always has an imaginary B and sometimes an E mode, whereas the C_4_ #2 structure, when it exists, usually has both. The C_2v_ structures all have an imaginary A_2_ mode. Most of the C_2v_ structures also have imaginary B_1_ and B_2_ modes. Usually, the C_2v_ #3 structure coalesces into the C_2v_ #1 structure. Desymmetrization of the C_4_ and C_2v_ structures leads to a plethora of C_2_ and C_s_ structures, many of which have imaginary modes and would desymmetrize to one of the many C_1_ structures. With one exception at one level, none of the C_2_ and C_s_ structures are 5-coordinated and lose at least one water to form 2-, 3-, and 4-coordinated structures. For the 6-31G* basis set (having no diffuse functions), in some cases, the chloride ion dissociates for some of the C_1_ structures, or one of the hydrogen atoms of the water molecules interacts with the copper atom instead of the chlorine atom. Notable local minimum energy structures (apart from the obvious C_1_ structures) include the C_2_ #2 [3 + 2], C_2_ #4 [3 + 2], C_s_ #5 [4 + 1], and C_s_ #7 [2 + 3].

**Figure 5:**
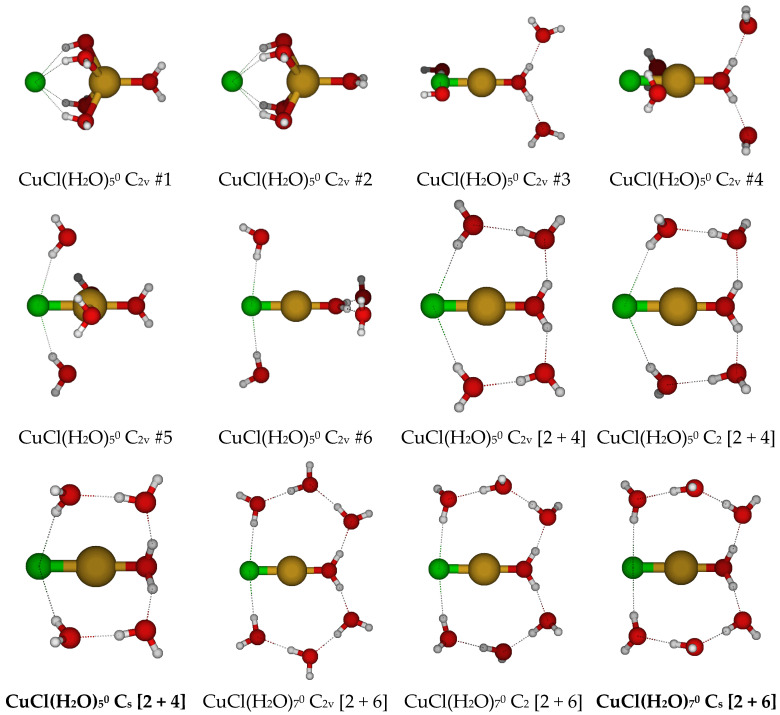
Chlorocopper(I) structures, CuCl(H_2_O)*_n_*^0^, *n* = 5, 7.

For CuCl(H_2_O)_5_^0^, we first considered six different C_2v_ structures in which the water molecules and chloride ion are directly bound to the copper(I) ion, initially either in the three σ_v_ planes or bisected by them (Figure 5). In every case, at least one ligand underwent dissociation from the copper(I) ion, and none of the resulting structures were local minima on the potential energy surface. Results using the 6-31G* basis set tended to undergo chloride dissociation, whereas those using basis sets with diffuse functions tended to undergo water dissociation.

We also examined dicoordinate CuCl(H_2_O)_7_^0^, starting with C_2v_ symmetry. These are desymmetrized to the unstable C_2_ and stable C_s_ structures.

To summarize, for monochloroaquacopper(I), a variety of 2-, 3-, and 4-coordinate structures can be formed, but the lowest-energy form seems to be the hydrated neutral dicoordinate [CuCl(H_2_O)]^0^ species. The results using the 6-31G* basis set were not typical of those using the 6-31+G* or 6-311+G* basis sets, with different energy orderings.

### 2.2. Structures of Dichloroaquacopper(I) Complexes, CuCl_2_(H_2_O)_n_^−^, n = 1–4, 6

**Figure 6:**
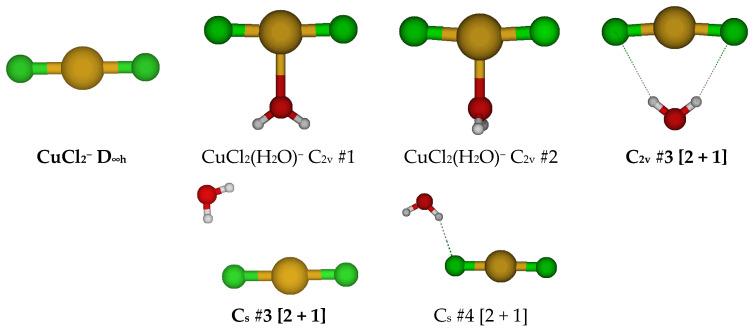
Dichlorocopper(I) structures, CuCl_2_(H_2_O)*_n_*^−^, *n* = 0, 1.

For CuCl_2_^−^, a linear D_∞h_ structure is obtained (see Figure 6). We may add a water molecule in one of several ways to give CuCl_2_(H_2_O)^−^. For those with a direct Cu-O interaction, we first consider one of the two possible C_2v_ structures. When both exist, C_2v_ #1 is always lower in energy. When it exists, the C_2v_ #2 has an imaginary A_2_ mode, whereas C_2v_ #1 does not. This suggests that the C_2v_ #2 would convert to the C_2v_ #1 structure along this mode. The C_2v_ #1 structure usually has imaginary B_1_ and B_2_ modes, suggesting desymmetrization to one of two C_s_ structures. Along the B_1_ mode, the C_2v_ #1 structure converts to the C_2v_ [2 + 1] structure via a C_s_ structure by the water wagging motion. Along the B_2_ mode, it converts to the stable C_s_ #3 [2 + 1]. There also exists a C_s_ #4 [2 + 1].

**Figure 7:**
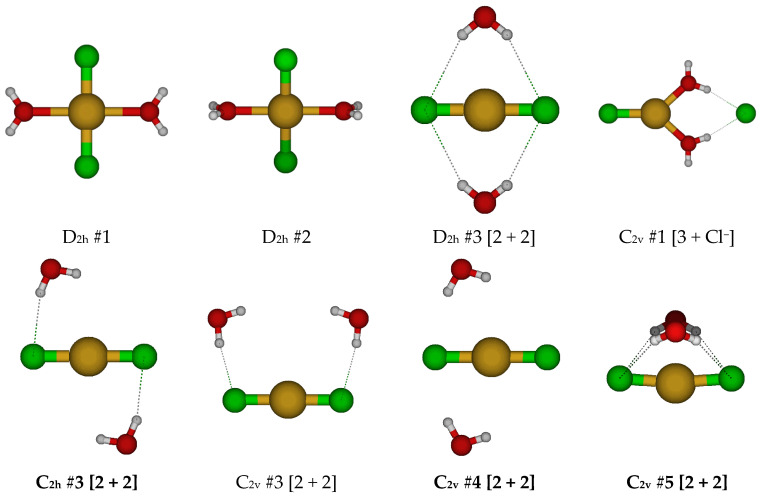

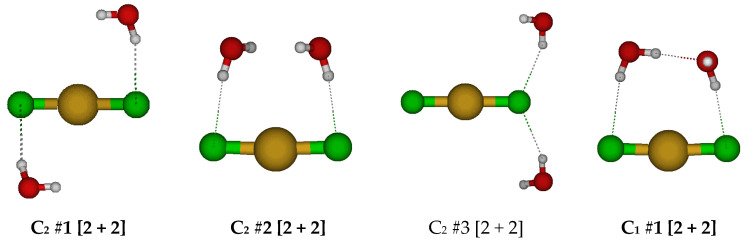
Dichlorocopper(I) structures, CuCl_2_(H_2_O)*_n_*^−^, *n* = 2.

For CuCl_2_(H_2_O)_2_^−^, we first examine the two possible D_2h_ square planar structures (Figure 7). For D_2h_ #2, the water molecules dissociated for all levels except B3LYP/6-31G*. The D_2h_ #1 structure has multiple imaginary frequencies of irreducible representation B_1g_, B_3g_, B_1u_, and B_3u_, whereas the D_2h_ #2 structure additionally has A_u_ and B_2g_ modes, which would convert via a D_2_ and C_2h_ structure to D_2h_ #1. Desymmetrization of the D_2h_ #1 structure along these modes would lead to C_2h_ #1, C_2h_ #2, C_2v_ #1, and C_2v_ #2 structures. For the C_2h_ #1 structure, the water wags to break coordination with the copper to form potentially a new D_2h_ #3 [2 + 2] structure. Desymmetrization along the ungerade B modes usually results in water detachment, except for B_3u_ at some HF levels, where a chloride detaches instead. For the D_2h_ #3 [2 + 2] structure, desymmetrization along the imaginary B_3g_, B_2u_, and B_3u_ modes would give C_2h_ #3, C_2v_ #3, and C_2v_ #4. These in turn desymmetrize to one of a number of C_i_, C_2_, and C_s_ structures, some of which are local minima at some levels of theory. The C_s_ structures often ascend in symmetry to C_2v_ #4 or C_2h_ #3. The lowest energy structure on the potential energy surface is the C_1_ #1 structure, in which a hydrogen bond is formed between the two water molecules. The only stable structures found may be described as hydrated CuCl_2_^−^ anions.

**Figure 8:**
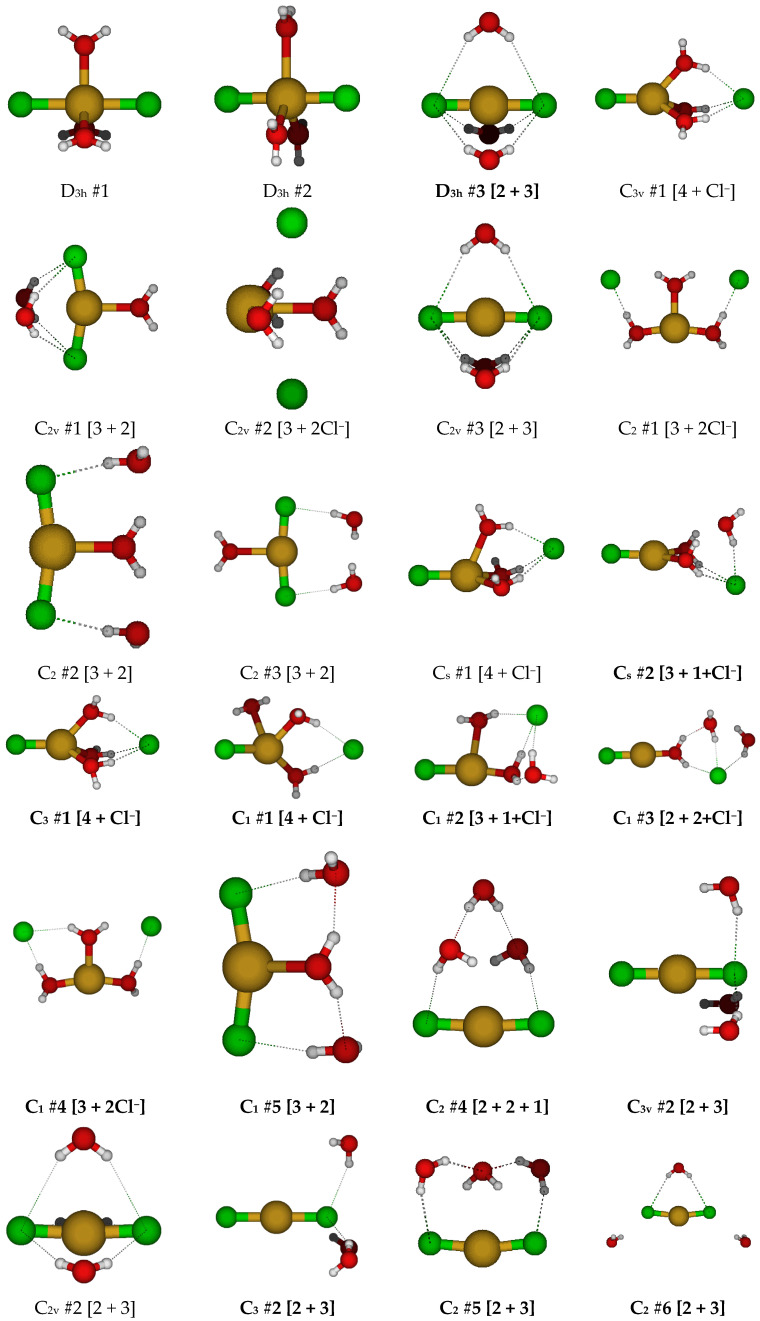

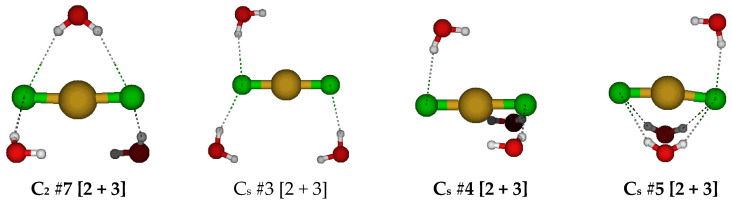
Dichlorocopper(I) structures, CuCl_2_(H_2_O)*_n_*^−^, *n* = 3.

For CuCl_2_(H_2_O)_3_^−^, we first examine the two possible D_3h_ trigonal bipyramidal structures (Figure 8). The first has the water molecules bisected by the σ_h_ symmetry plane, whereas the second has the water molecules in the σ_h_ symmetry plane. In the second case, for diffuse basis sets, the water molecules dissociated, whereas for the 6-31G* basis set, the structures have imaginary E”, A_2_”, and A_1_” modes, and at the correlated levels, additional A_2_’ and E’ modes. In the first case, there are imaginary A_2_’, E’, E”, and A_2_” modes at all levels of theory. For D_3h_ #1, desymmetrization along A_2_’ to give C_3h_ #1 resulted in ascent in symmetry via water wagging to form D_3h_ #3 [2 + 3], which was stable at HF/6-31G*. Desymmetrization along A_2_” gave the C_3v_ #1 structure in which one of the chlorine atoms dissociated. Desymmetrization along the E’ mode gave one of two possible C_2v_ structures, one (C_2v_ #1, most levels of theory) in which two water molecules wagged to form hydrogen bonds with the chlorine atoms, or one (C_2v_ #2, B3LYP/6-31G* and MP2/6-31G*) in which the two chlorine atoms dissociated. The three C_2_ structures found either have two dissociated chlorine atoms (C_2_ #1) or two dissociated water molecules (C_2_ #2, #3). The two C_s_ structures, both (C_s_ #1 and #2), have a dissociated Cl^−^; in the second one, there is also a dissociated water. The C_s_ #2 structure was stable at the Hartree–Fock levels. We did not explore the desymmetrization of D_3h_ #2, since it dissociated at all levels with diffuse functions.

The C_3v_ #1 structure had imaginary A_2_ and E modes at all levels of theory. Desymmetrization along the A_2_ mode would give a C_3_ structure, which is stable at some levels of theory. Desymmetrizing to give C_1_ structures often resulted in a wide variety of atomic arrangements. While desymmetrization from a maximally coordinated structure does prove that lower coordination numbers are favored, it is tedious and may not be the best method to find all structures, so we also try desymmetrizing from the high symmetry D_3h_ #3 trihydrated dichlorocuprate(I) ion.

The D_3h_ #3 structure usually contained imaginary A_2_” and E” modes (and occasionally E’ modes). The resulting C_2v_ #3 structure always has imaginary A_2_ and B_2_ (and sometimes B_1_) modes. The resulting C_3v_ #2 structure was not stable (except at MP2/6-31G*) and contained imaginary A_2_ and/or E modes. The C_2v_ #3 structure can desymmetrize to a number of C_2_ and C_s_ structures, of which C_2_ #5 is stable at HF/6-31G*, HF/6-311+G* and MP2/6-311+G*; C_2_ #7 is stable at B3LYP/6-31G*, MP2/6-31G*, and MP2/6-311+G*; C_s_ #4 is stable at B3LYP/6-31+G* and B3LYP/6-311+G*; and C_s_ #5 is stable at MP2/6-31+G* and MP2/6-311+G*.

**Figure 9:**
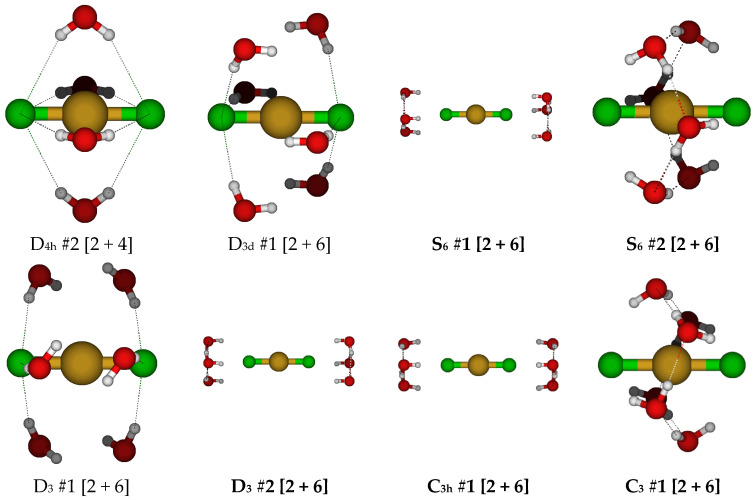
Dichlorocopper(I) structures, CuCl_2_(H_2_O)*_n_*^−^, *n* = 4, 6.

For copper(I) with two chloride ions, it appears that five-coordination was not possible, and the lowest energy structures appeared to be hydrated dicoordinate dichlorocuprate(I), so initial structures with coordination numbers higher than this were not expected to be stable. At this point, we focused on structures having a discrete CuCl_2_^−^ anion with six water molecules. We started with the D_3d_ #1 structure, which had imaginary A_2g_, A_1u_, and usually E_g_ and E_u_ modes (see Figure 9). The Hartree–Fock structures had the water molecules farther away from the copper ion by increasing the H…Cl-Cu angle. The resulting S_6_ #1, D_3_ #2, and C_3h_ #1 structures were quite close in energy, and at least one of them was a local minimum and the lowest in energy at the HF levels. The S_6_ #2 structure was the lowest in energy at the MP2 levels, and the D_3_ #2 structure was the lowest in energy at the B3LYP levels. The D_3_ #1 structure desymmetrized to give C_3_ #1, which ascended in symmetry at some HF levels to S_6_ #2.

### 2.3. Structures of Trichloroaquacopper(I) Complexes, CuCl_3_(H_2_O)_n_^2−^, n = 1–4, 6

**Figure 10:**
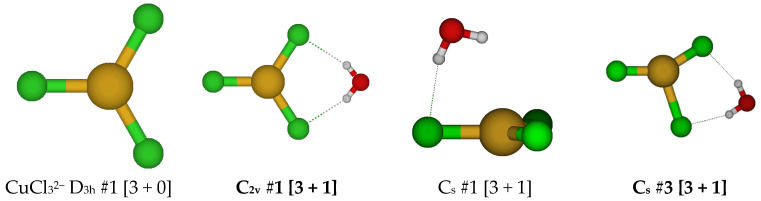
Trichlorocopper(I) structures, CuCl_3_(H_2_O)*_n_*^2−^, *n* = 0, 1.

For CuCl_3_^2−^, a trigonal planar D_3h_ structure is obtained (see Figure 10). We may add a water molecule in one of two ways (C_s_ #1, C_s_ #2) to make a tetrahedrally coordinated structure; however, the water molecule always dissociates. For C_s_ #2, the structure ascends in symmetry to give a C_2v_ #1 [3 + 1] structure. The dissociated structure for C_s_ #1 is a transition state for water migration between two equivalent C_2v_ #1 structures in which only one hydrogen bond is retained. Other attempts to make tetrahedrally coordinated structures led to structures such as CuCl_2_^−^ + H_2_O + Cl^−^, CuCl_2_(H_2_O)^−^ + Cl^−^, or CuCl(H_2_O) + 2 Cl^−^.

**Figure 11:**
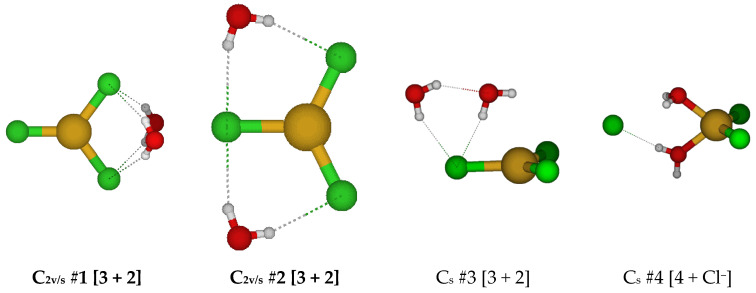
Trichlorocopper(I) structures, CuCl_3_(H_2_O)*_n_*^2−^, *n* = 2.

For CuCl_3_(H_2_O)_2_^2−^, all attempts to construct a five-coordinate structure led to dissociation of either water or chloride ligand(s). Two stable dihydrated trichlorocuprate(I) structures were found (Figure 11).

**Figure 12:**
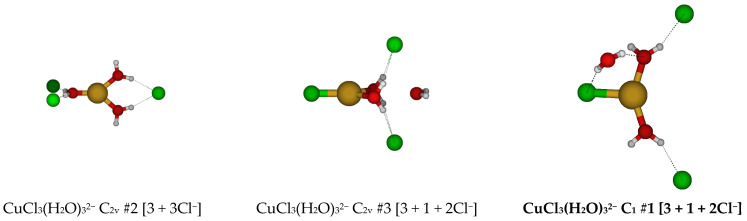

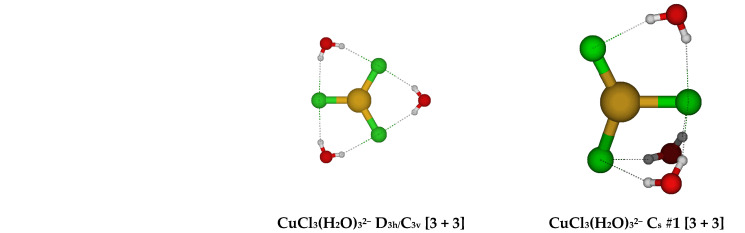
Trichlorocopper(I) structures, CuCl_3_(H_2_O)*_n_*^2−^, *n* = 3.

For CuCl_3_(H_2_O)_3_^2−^, all attempts to construct a six-coordinate structure failed, resulting in dissociation of chloride and/or water ligands. Two possible trihydrated trichlorocuprate(I) ions were identified, of D_3h_ (C_3v_ at MP2 levels) and C_s_ symmetry (Figure 12). At the MP2/6-31G* level, the most hydrogen-bonded chloride ion of the C_s_ #1 structure dissociated.

**Figure 13:**
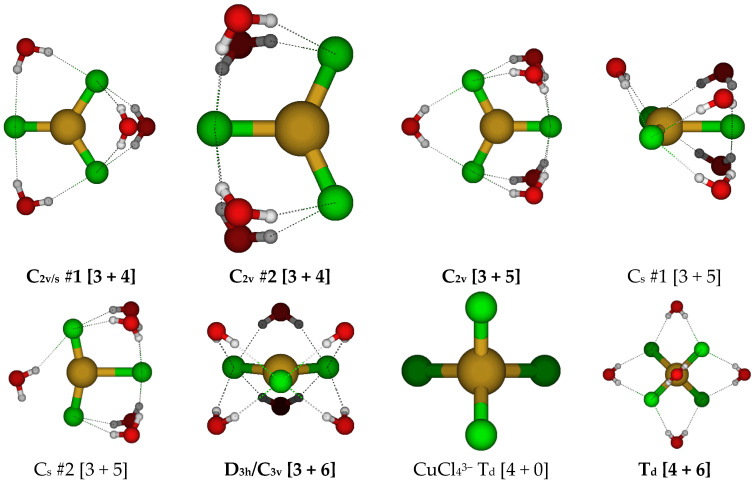
Trichlorocopper(I), CuCl_3_(H_2_O)*_n_*^2−^, *n* = 4–6, and tetrachlorocopper(I) CuCl_4_(H_2_O)*_n_*^3−^, *n* = 0, 6 structures.

At this point, it was decided to focus on hydrated trichlorocuprate(I) ions (Figure 13). With four water molecules, there are two main arrangements that can be considered, with C_2v_ #1 being slightly more stable than C_2v_ #2. With five water molecules, the main arrangement is the C_2v_ structure. For six water molecules, we return to a D_3h_ structure. At some levels of theory, slightly lower symmetry stationary points may be located.

### 2.4. Structures of Tetrachloroaquacopper(I) Complexes, CuCl_4_(H_2_O)_n_^3−^, n = 0, 6

For the putative tetrachlorocuprate(I) ion, we only considered two structures, the naked ion and the hexahydrate (Figure 13). The naked ion nearly always has an imaginary T_2_ mode corresponding to Cu-Cl stretching, suggesting fragmentation of this highly charged ion. However, to our surprise, hydrating this ion with six water molecules results in stabilization of this structure to a local minimum at most levels of theory.

Because of the anomalous structures calculated using the 6-31G* basis set, we do not consider these calculations further.

## 3. Discussion

In the solid state under ambient conditions, CuCl(s) adopts the sphalerite (zinc-blende) structure (CuCl-II, F-43m), with the tetrahedrally coordinated Cu-Cl distance of 2.382 Å [6], later refined to 2.3407 Å [7] and 2.347 Å [8]. It transforms to the hexagonal wurtzite structure CuCl-I at 681 K before melting at 696 K [9]. In the wurtzite structure, the two Cu-Cl distances are 2.3751 and 2.4717 Å. Above a triple point (1.2 kbar, 724 K), both of the forms transform to the body-centred cubic form CuCl-III (P6_3_mc) upon temperature increase. Increasing the pressure at ambient temperature converts the sphalerite form to CuCl-IV (Pa-3, ~50 kbar), then CuCl-V (~100 kbar) [10]. For CuCl-IV, the Cu-Cl distances are 2.2922, 2.4182 Å (5.52 GPa) and 2.2562, 2.3674 Å (92.4 Gpa), whereas for CuCl-V (halite, Fm-3m), the Cu-Cl distances are 2.4645 Å around these octahedrally coordinated atoms.

In the gas phase, CuCl(g) can be generated by passing HCl(g) over hot Cu(s) metal at 800–1000 °C. Brewer and Lofgren established that the vapor consists of a mixture of CuCl(g) and its trimer Cu_3_Cl_3_(g) based on vapor pressure and density measurements [11]. No evidence of the dimer was found. The trimer has a single infrared band at 350 cm^−1^ [12], whereas the monomer occurs at 416.9 cm^−1^ [13]. This is consistent with a cyclic structure. The electron diffraction results of Wong and Schomaker fit a planar cyclic model with Cu-Cl = 2.160(15) Å and Cu-Cl-Cu = 87.5° [14]. In the near-ultraviolet, absorbances at 223.5, 218(sh), 238(sh), and 273 nm were observed [15]. Our calculations support a stable D_3h_ trimer structure (Figure 1).

The Cu-Cl distance in select [CuCl(H_2_O)_n_]^0^ structures is given in Table 1. At all levels of theory, the Cu-Cl distance shortens slightly as the first water molecule is added, but then is generally lengthened as more water molecules hydrate the resulting [CuCl(H_2_O)]^0^ molecule. The Cu-Cl distance in the unhydrated molecule (2.10 Å, B3LYP/6-311+G*) is much shorter than in the solid state (2.25–2.47 Å, above) but is only slightly shorter than that reported in the gas-phase for the trimer (2.16 Å, above). The predicted trimer Cu-Cl distance is very close (2.1494 Å, MP2/6-311+G*). In the solid state, each copper atom is surrounded by between 4 and 6 chlorine atoms, whereas in the gas-phase monomer, only one atom is nearby, which is why the distance is shorter in the monomer.

The Cu-Cl vibrational frequencies in select [CuCl(H_2_O)_n_]^0^ structures are given in Table 2. The experimental harmonic value in CuCl(g) is given as 416.9 cm^−1^, which matches well with the MP2/6-311+G* value of 414 cm^−1^. For the monohydrate, the Cu-Cl stretching motion couples with the Cu-O stretching motion in a symmetric low-frequency and an asymmetric high-frequency pair. The lower frequency of the hydrated forms of this linear species is only slightly affected by the hydration number, whereas the higher frequency component is significantly affected. A weak band observed at 350 cm^−1^ in copper(I) chloride solutions, assigned to CuCl^0^, is in good agreement with the predicted band for hexahydrated [CuCl(H_2_O)]^0^ at 365 cm^−1^ (MP2/6-311+G*).

The discrete dichlorocuprate(I) [CuCl_2_]^−^ ion is found in the tetraphenylarsonium/phosphonium dichlorocuprate(I) salts, among others [16]. In these the CuCl distances are: [Ph_4_As]^+^[CuCl_2_]^−^, 2.069(3) and 2.072(3) Å; [Ph_4_P]^+^[CuCl_2_]^−^, 2.088(2) and 2.090(2) Å. In solution (acetonitrile, dimethyl sulfoxide, pyridine, water), the distances were found to be 2.10(2)–2.12(2) by EXAFS, which demonstrates that the same ion is present [17]. These solid-state and solution distances compare quite favorably to the gas-phase MP2 calculations on the unsolvated dichlorocuprate(I) ion of ~2.10 Å (Table 3). In most of the hydrated structures, the Cu-Cl distance is hardly affected by the hydration, except for the S_6_ #2 structure, which has a much longer Cu-Cl distance.

The infrared and Raman spectra of solutions and solids containing the dichlorocuprate(I) ion were summarized by Bowmaker et al., who found that in the infrared, a band around 400 cm^−1^ appears, whereas in the Raman, a band around 300 cm^−1^ appears [18]. These can be assigned to the antisymmetric and symmetric stretching motions, respectively. In addition, an IR band at 110 cm^−1^ corresponds to the bending mode. More specifically, in ether extracts of HCl + CuCl, a band occurs at 296 cm^−1^; in tributylphosphate extracts of HCl + CuCl, at 397 and 301 cm^−1^; in tributylphosphate solution of CuCl + LiCl, at 405, 109, and 300 cm^−1^; in polycrystalline tetrabutylammonium dichlorocuprate(I), at 404, 111, and 304 cm^−1^; and in polycrystalline tetraphenylarsonium dichlorocuprate(I), at 408, 111, and 315 cm^−1^. The mutual exclusion rule between the IR and Raman spectra for centrosymmetric moieties such as [CuCl_2_]^−^ can clearly be seen. In aqueous solution, we found a band attributable to [CuCl_2_]^−^ at 297 cm^−1^ [4]. Both the MP2 and B3LYP calculations give good agreement with these experiments, with the MP2 calculations being slightly too high and the B3LYP calculations slightly too low (Table 4). The Hartree–Fock calculations severely underestimate the vibrational frequencies, which is atypical because usually Hartree–Fock frequencies are larger than experimental frequencies and are typically scaled by a factor of around 0.9 [19]. The effect of the water molecules upon hydration is to increase the frequency of the stretching modes by 3-14 cm^−1^. In some cases, coupling between the antisymmetric stretching mode and water librations results in two modes with an appreciable contribution of Cu-Cl stretch (n = 6). The hexahydrate S_6_ #2 structure has a much longer Cu-Cl distance, resulting in much smaller frequencies.

The discrete trichlorocuprate ion was found in bis(tetramethylphosphonium) trichlorocuprate(I) salt [20]. The Cu-Cl distances were reported as 2.214(2) and 2.232(3) Å. In aqueous solution, EXAFS gives 2.20(2) or 2.21(3) Å, depending on the analysis [17]. These values are in fair agreement with the MP2 calculations of the hexahydrate shown in Table 5, but the HF and B3LYP calculations are much too long. The general trend is that there is a significant shortening of the Cu-Cl distance on hydration, which suggests a strong stabilization of this small, highly charged ion. For some structures (3, C_s_; 4 C_2v_ #2), a pronounced alternation of Cu-Cl bonds occurs as the stabilization by hydrogen bonding results in partial dissociation.

The increase in the Cu-Cl stretching vibrational frequencies of CuCl_3_^2−^ upon hydration is also pronounced, concomitant with the shortening of the Cu-Cl bonds. In the two cases where the Cu-Cl bond lengthened, one of the vibrational frequencies significantly decreased. Experimentally, we observed a Raman band at 247 cm^−1^ that we assigned to the Cu-Cl stretching motion of the CuCl_3_^2−^ ion. This is in fair agreement with the totally symmetric band predicted at 235 cm^−1^ (MP2/6-31+G*, Table 6). We also noticed that at some levels of theory, the predicted symmetry was lower than anticipated, but the calculations of the bond distances and vibrational frequencies at higher symmetry, containing an imaginary mode, were only slightly altered by the lower symmetry.

We were initially unable to find any experimental literature reports of a discrete CuCl_4_^3−^ anion. Our calculations on the tetrahedrally constrained ion gave long Cu-Cl distances, which shortened considerably upon hydration (Table 7). Although the unhydrated ion was unstable with respect to fragmentation (see the imaginary frequencies in Table 8), the addition of water molecules to form a highly symmetric T_d_ structure stabilized the structure so that it could form a local minimum. In the gas phase, the formation of CuCl_3_^2−^ and CuCl_4_^3−^ by the addition of chloride to the precursor anion would be thermodynamically unfavored. However, the hydration energy would tend to stabilize the highly charged anions in aqueous solutions.

A search of the Cambridge Structural Database revealed 274 structures containing the [CuCl_2_]- anion, with an average bond distance of 2.096 Å and bond angle of 178° [21]. No hydrated forms of this ion were found, i.e., neither [CuCl_2_(H_2_O)]^−^ nor [CuCl_2_(H_2_O)_2_]^−^, only those of the corresponding copper(II) ion (identifiers ZUKTUX, FUTRUH, KUPHAE, RITYUR). In addition, 24 structures containing the [CuCl_3_] anion were found, most of which were the [CuCl_3_]^2−^ anion (Cu-Cl avg 2.243 Å), except for the three instances of the [CuCl_3_]^−^ anion with copper(II) (identifiers INAYAZ, GEXRIK, MILQIH) and much shorter Cu-Cl distances. Although the average angle is 119.9(6.8)° and the sum 359.6(1.1)°, as expected for a trigonal planar structure, the large standard deviation of 6.8° means that there are some significant in-plane distortions. The 15 [CuCl_3_(H_2_O)] and 9 [CuCl_3_(H_2_O)_2_] anions found all had copper in the +2 oxidation state and an overall charge of -1. Of the 744 structures containing [CuCl_4_], the vast majority contained copper(II) as the [CuCl_4_]^2−^ ion. These structures were statistically analyzed to find outliers (at least one Cu-Cl distance more than two standard deviations from the average of 2.257(0.031) Å), and these were manually checked. We found 66 such structures, but again the vast majority of these were the [CuCl_4_]^2−^ ion, and only three instances of the [CuCl_4_]^3−^ ion were found (identifiers FAMQAL (2.376(0) Å), JETRIN (2.338(0.018) Å), and LADREP (2.202(0.043) Å)), in which all four of the CuCl distances were more than two standard deviations from the average. The last of these is of poor quality due to disorder. Only the tetrachlorocopper(II) structures could also coordinate to one (identifiers COCNEO, COGXOP, HOWCOL, VEWQEX) or two (identifiers DEVVUZ, GADWUD, HERWEK) water molecules. The fact that the di-, tri-, and tetrachlorocopper(I) anions do not directly bind to water molecules supports our computational findings and indicates that the copper(I) ion prefers to bind to the chloride ion rather than the water molecule.

## 4. Materials and Methods

Preliminary calculations were performed using Gaussian 98 [22]. The MP2 calculations utilize the frozen core approximation. The geometries were optimized using a stepping-stone approach, in which the geometries at the levels HF/STO-3G, HF/3-21G, HF/6-31G*, HF/6-31+G*, MP2/6-31G*, and MP2/6-31+G* were sequentially optimized, with the geometry and molecular orbital reused for the subsequent level. When this approach failed (as one or more ligands dissociated or underwent pseudorotation), the problematic level was skipped. Default optimization specifications were normally used. After each level, where possible, a frequency calculation was performed at the same level, and the resulting Hessian was used in the following optimization. Z-matrix coordinates constrained to the appropriate symmetry were used as required to speed up the optimizations. Because frequency calculations are done at each level, any problems with the Z-matrix coordinates would manifest themselves by giving imaginary frequencies corresponding to modes orthogonal to the spanned Z-matrix space. The Hessian was evaluated at the first geometry (opt = CalcFC) for the first level in a series to aid geometry convergence. For structures that proved not to be local minima at the HF/6-31G* level, the subsequent calculations were not performed.

The calculated structures proved to be very sensitive to the choice of theoretical level, with large changes in optimum geometry sometimes between two sequential calculations. In addition, calculations using the low-level basis sets often converged to incorrect copper(I) ion configurations (s^2^d^8^ or s^1^d^9^ instead of d^10^) that were propagated throughout the calculations, as demonstrated by wavefunction stability calculations. It was decided to repeat all calculations starting with the highest possible symmetry and systematically desymmetrizing (Table 9) at all appropriate levels of theory, skipping the STO-3G and 3-21G levels, and using either Gaussian 03 [23] or Gaussian 16 [24]. In addition, the use of the 6-311+G* basis set and the B3LYP functional was explored.

## 5. Conclusions

The speciation of copper(I) with increasing amounts of chloride is consistent with the existence of [CuCl(H_2_O)], [CuCl_2_]^−^, and [CuCl_3_]^2−^. The addition of explicit waters of hydration has been shown to improve the agreement with the experimental solution vibrational frequencies. The MP2 method has been shown to give good results for the geometries and vibrational frequencies of chlorocopper(I) complexes when used with basis sets including diffuse functions. Systematic desymmetrization has proven to be advantageous in finding structures and/or ruling out candidate structures.

## Figures and Tables

**Table 1:** Cu-Cl distance (Å) in [CuCl(H_2_O)_n_]^0^. In this and the following tables, n/a = not applicable.

Species (n, Label)	HF		B3LYP		MP2	
	6-31+G*	6-311+G*	6-31+G*	6-311+G*	6-31+G*	6-311+G*
0, C_∞v_	2.1746	2.1834	2.0937	2.1023	2.0550	2.0638
0, Cu_3_Cl_3_, D_3h_	2.3007	2.3098	2.2067	2.2120	2.1419	2.1494
1, C_s_	2.1713	2.1809	2.0922	2.0992	2.0422	2.0484
2, C_1_ #1 [2 + 1]	2.1749	2.1865	2.0975	2.1054	2.0458	2.0528
2, C_1_ #2 [2 + 1]	2.2084	2.2226	2.0972	2.1049	n/a	n/a
3, C_1_ #3 [2 + 2]	2.2001	2.2135	2.1181	2.1256	2.0636	2.0712
4, C_1_ #4 [2 + 2 + 1]	2.1987	2.2123	2.1189	2.1273	2.0642	2.0725
5, C_s_ #1 [2 + 4]	2.2247	2.2416	2.1341	2.1427	2.0767	2.0849
7, C_s_ #1 [2 + 6]	2.2253	2.2416	2.1401	2.1496	2.0812	2.0911

**Table 2:** Cu-Cl(O) stretching vibrational frequency (cm^−1^) in [CuCl(H_2_O)_n_]^0^.

Species (n, Label)	HF		B3LYP		MP2	
	6-31+G*	6-311+G*	6-31+G*	6-311+G*	6-31+G*	6-311+G*
0, C_∞v_	357	355	394	388	422	414
0, Cu_3_Cl_3_, D_3h_	207, 239, 273, 300	210, 238, 276, 301	214, 294, 299, 368	213, 296, 302, 371	241, 335, 345, 417	235, 326, 340, 410
1, C_s_	260, 379	263, 380	327, 438	334, 446	362, 487	370, 490
2, C_1_ #1 [2 + 1]	296, 390	298, 394	343, 458	354, 468	388, 505	386, 513
2, C_1_ #2 [2 + 1]	276, 347	282, 345	341, 457	355, 469	n/a	n/a
3, C_1_ #3 [2 + 2]	284, 377	285, 379	342, 459	341, 467	373, 495	370, 499
4, C_1_ #4 [2 + 2 + 1]	275, 391	280, 395	336, 484	337, 491	386, 513	382, 520
5, C_s_ #1 [2 + 4]	279, 392	279, 396	335, 483	331, 486	375, 525	364, 533
7, C_s_ #1 [2 + 6]	284, 415	280, 422	336, 513	334, 514	375, 553	365, 559

**Table 3:** Cu-Cl distance (Å) in [CuCl_2_(H_2_O)_n_]^−^.

Species (n, Label)	HF		B3LYP		MP2	
	6-31+G*	6-311+G*	6-31+G*	6-311+G*	6-31+G*	6-311+G*
0, D_∞h_	2.2498	2.2620	2.1640	2.1709	2.0992	2.1059
1, C_s_ #1	2.257, 2.241	2.270, 2.253	2.167, 2.154	2.175, 2.161	2.102, 2.091	2.1025
2, C_1_ #1	2.256, 2.238	2.269, 2.250	2.168, 2.153	2.176, 2.160	2.103, 2.092	2.111, 2.098
3, C_2_ #4	n/a	n/a	2.1597	n/a	2.0929	n/a
3, C_2_ #5	n/a	2.2561	n/a	n/a	n/a	2.1022
3, C_2_ #6	n/a	n/a	n/a	n/a	n/a	2.0985
3, C_s_ #4	n/a	n/a	2.163, 2.152	2.172, 2.159	n/a	n/a
6, S_6_ #1	2.2557	2.2688	2.1620	2.1692	2.0970	2.1048
6, S_6_ #2	2.3630	2.3937	2.2516	2.2665	2.1674	2.1811
6, D_3_ #2	2.2557	2.2688	2.1620	2.1692	2.0970	2.1048
6, C_3h_ #1	2.2558	n/a	2.1620	2.1692	2.0970	n/a

**Table 4:** Cu-Cl stretching vibrational frequency (cm^−1^) in [CuCl_2_(H_2_O)_n_]^−^.

Species (n, Label)	HF		B3LYP		MP2	
	6-31+G*	6-311+G*	6-31+G*	6-311+G*	6-31+G*	6-311+G*
0, D_∞h_	234, 332	232, 332	274, 376	271, 376	314, 432	305, 425
1, C_s_ #1	232, 334	233, 332	278, 383	272, 384	319, 436	311, 431
2, C_1_ #1	237, 336	234, 334	278, 383	275, 383	315, 436	306, 429
3, C_2_ #4	n/a	n/a	278, 384	n/a	323, 442	n/a
3, C_2_ #5	n/a	242, 334	n/a	n/a	n/a	312, 436
3, C_2_ #6	n/a	n/a	n/a	n/a	n/a	318, 437
3, C_s_ #4	n/a	n/a	287, 387	284, 387	n/a	n/a
6, S_6_ #1	243, 320/347	240, 313/341	286, 347/389	284, 346/390	318, 438	314, 430
6, S_6_ #2	202, 263	198, 253	240, 315	237, 312	274, 375	263, 364
6, D_3_ #2	243, 320/347	240, 313/342	286, 390	284, 390	318, 438	314, 430
6, C_3h_ #1	243, 319/347	n/a	286, 390	284, 390	318, 438	n/a

**Table 5:** Cu-Cl distance (Å) in [CuCl_3_(H_2_O)_n_]^2−^.

Species (n, Label)	HF		B3LYP		MP2	
	6-31+G*	6-311+G*	6-31+G*	6-311+G*	6-31+G*	6-311+G*
0, D_3h_	2.5402	2.5440	2.4035	2.4093	2.3019	2.3243
1, C_2v_	2.479, 2.547	2.487, 2.548	2.343, 2.416	2.354, 2.419	2.262, 2.309	2.282, 2.333
2, C_2v_ #1	2.480, 2.563	2.487, 2.558	2.346, 2.448	2.356, 2.447	2.267, 2.322	2.287, 2.348
2, C_s_ #1	n/a	n/a	n/a	n/a	2.266, 2.326	2.285, 2.351
2, C_2v_ #2	2.431, 2.559	2.442, 2.554	2.301, 2.433	2.313, 2.433	2.230, 2.320	2.245, 2.340
3, D_3h_	2.489	2.4905	2.3670	2.3717	2.2761	2.2967
3, C_3v_	n/a	n/a	n/a	n/a	2.2757	2.2966
3, C_s_	2.431, 2.484, 2.584	2.441, 2.486, 2.573	2.297, 2.341, 2.511	2.309, 2.350, 2.495	2.231, 2.270, 2.348	2.248, 2.286, 2.368
4, C_2v_ #1	2.437, 2.498	2.443, 2.495	2.315, 2.384	2.324, 2.384	2.238, 2.288	2.257, 2.306
4, C_s_ #1	n/a	n/a	n/a	n/a	2.237, 2.289	2.258, 2.304
4, C_2v_ #2	2.433, 2.609	2.440, 2.591	2.284, 2.622	2.300, 2.567	2.237, 2.366	2.255, 2.370
5, C_2v_	2.444, 2.509	2.446, 2.500	2.326, 2.412	2.332, 2.403	2.166, 2.565	2.268, 2.307
6, D_3h_	2.4513	2.4494	2.3446	2.3450	2.2587	2.2734
6, C_3v_	n/a	n/a	n/a	n/a	n/a	2.2733

**Table 6:** Cu-Cl stretching vibrational frequency (cm^−1^) in [CuCl_3_(H_2_O)_n_]^2−^. The “*” indicates that the structure was not an energy minimum.

Species (n, Label)	HF		B3LYP		MP2	
	6-31+G*	6-311+G*	6-31+G*	6-311+G*	6-31+G*	6-311+G*
0, D_3h_	125, 144	130, 146	152, 172	160, 175	207, 206	198, 199
1, C_2v_	120, 143, 158	126, 147, 161	145, 174, 202	148, 175, 199	197, 209, 238	187, 202, 229
2, C_2v_ #1	124, 159, 163	132, 162, 168	142, 191, 204	151, 192, 209	198, 223, 245 *	189, 215, 237 *
2, C_s_ #1	n/a	n/a	n/a	n/a	196, 224, 247	187, 215, 238
2, C_2v_ #2	116, 148, 180	121, 152, 183	132, 175, 225	137, 178, 226	185, 210, 264	178, 206, 257
3, D_3h_	155, 163	162, 167	184, 189	190, 192	223, 233 *	216, 224 *
3, C_3v_	n/a	n/a	n/a	n/a	223, 234	216, 225
3, C_s_	119, 161, 185	127, 165, 189	120, 197, 235	129, 199, 236	185, 227, 270	179, 220, 262
4, C_2v_ #1	147, 164, 181	157, 170, 186	172, 190, 220	180, 196, 222	220, 225, 262 *	213, 219, 253 *
4, C_s_ #1	n/a	n/a	n/a	n/a	220, 225, 262	214, 219, 252
4, C_2v_ #2	116, 171, 196	125, 174, 201	102, 212, 262	111, 210, 260	179, 236, 280	180, 228, 269
5, C_2v_	153, 174, 186	163, 179, 193	173, 204, 223	181, 207, 229	220, 238, 264	218, 231, 253
6, D_3h_	177, 178	182, 187	204, 205	208, 214	235, 252	229, 245 *
6, C_3v_	n/a	n/a	n/a	n/a	n/a	229, 244

**Table 7:** Cu-Cl distance (Å) in [CuCl_4_(H_2_O)_n_]^3−^.

Species (n, Label)	HF		B3LYP		MP2	
	6-31+G*	6-311+G*	6-31+G*	6-311+G*	6-31+G*	6-311+G*
0, T_d_	3.0931	3.1508	2.8015	2.8246	2.5907	2.6269
6, T_d_	2.7513	2.7443	2.5760	2.5652	2.4450	2.4663

**Table 8:** Cu-Cl stretching vibrational frequency (cm^−1^) in [CuCl_4_(H_2_O)_n_]^3−^.

Species (n, Label)	HF		B3LYP		MP2	
	6-31+G*	6-311+G*	6-31+G*	6-311+G*	6-31+G*	6-311+G*
0, T_d_	85i,61	87i,55	78i,88	91i,79	56i,88	64i,88
6, T_d_	45,122	41,122	78,137	85,143	120,166	119,164

**Table 9:** Desymmetrization table (adapted from supplementary material of Ref. [5]). Desymmetrization of a structure with symmetry of a point group along an imaginary mode of irreducible representation given will give a structure with symmetry of a subgroup.

Order	Point Group	Irreducible Representation	Subgroup
2	C_s_	A”	C_1_
	C_i_	A_u_	C_1_
	C_2_	B	C_1_
3, 5, 7	C_3/5/7_	E_n_	C_1_
4	C_4_	B	C_2_
		E	C_1_
	D_2_	B_1/2/3_	C_2_ (z/y/x)
	C_2v_	A_2_	C_2_
		B_1/2_	C_s_ (σ_v_(xz)/σ_v_(yz))
	C_2h_	B_g_	C_i_
		A_u_	C_2_
		B_u_	C_s_
	S_4_	B	C_2_
		E	C_1_
6	C_6_	B	C_3_
		E_1_	C_1_
		E_2_	C_2_
	D_3_	A_2_	C_3_
		E	C_2_
	C_3v_	A_2_	C_3_
		E	C_s_
	C_3h_	E’	C_s_
		A”	C_3_
		E”	C_1_
	S_6_	E_g_	C_i_
		A_u_	C_3_
		E_u_	C_1_
	T	E	D_2_
		T	C_3_
8	C_8_	B	C_4_
		E_1/3_	C_1_
		E_2_	C_2_
	D_4_	A_2_	C_4_
		B_1/2_	D_2_ (C_2_’/C_2_”)
		E	C_2_ (C_2_’ or C_2_”)
	C_4v_	A_2_	C_4_
		B_1/2_	C_2v_ (σ_v/d_)
		E	C_s_ (σ_v/d_)
	C_4h_	B_g_	C_2h_
		E_g_	C_i_
		A_u_	C_4_
		B_u_	S_4_
		E_u_	C_s_
	D_2h_	B_1/2/3g_	C_2h_ (C_2_(z/y/x))
		A_u_	D_2_
		B_1/2/3u_	C_2v_ (C_2_(z/y/x))
	D_2d_	A_2_	S_4_
		B_1_	D_2_
		B_2_	C_2v_
		E	C_2_ (C_2_’) or C_s_
	S_8_	B	C_4_
		E_1/3_	C_1_
		E_2_	C_2_
12	D_3h_	A_2_’	C_3h_
		E’	C_2v_
		A_1_”	D_3_
		A_2_”	C_3v_
		E”	C_2_ or C_s_ (σ_v_)
	D_3d_	A_2g_	S_6_
		E_g_	C_2h_
		A_1u_	D_3_
		A_2u_	C_3v_
		E_u_	C_2_ or C_s_

## Data Availability

The original contributions presented in this study are included in the article/Supplementary Material. Further inquiries can be directed to the corresponding author.

## Supplementary Materials

The following supporting information can be downloaded at: https://www.mdpi.com/article/10.3390/molecules30153147/s1, Table S1: Total Energies.

## Abbreviations

The following abbreviations are used in this manuscript:

HF	Hartree–Fock
MP2	Second-order Moller–Plesset Perturbation Theory
B3LYP	Becke three-parameter exchange with Lee–Yang–Parr correlation functional
