# Supplementary material for: An Ab Initio Study of Aqueous Copper(I) Speciation in the Presence of Chloride
Source: Molecules. 2025 Jul 27;30(15):3147. doi: 10.3390/molecules30153147 (PMC12348342; doi:10.3390/molecules30153147)
Supplement: Supplementary file 1 [file molecules-30-03147-s001.zip › molecules-3746818-supplementary.pdf]

Supplementary Material:
An Ab Initio Study of Aqueous Copper(I) Speciation in the Presence of Chloride

Daniel C. M. Whynot, Christopher R. Corbeil, Darren J. W. Mercer and Cory C. Pye *

Department of Chemistry, Saint Mary's University, 923 Robie Street, Halifax, NS B3H 3C3, Canada

Table S1. Total Energies (Bold indicates a local minimum)

	HF/STO-3G	HF/3-21G
Cu^+ K_h	(d^8s^2) **-1620.2204868**<br>**-1620.0889276**	(d^9s^1) **-1630.6760974**<br>**-1630.4158906**<br>**-1630.5361396**
H_2O C_{2v}	**-74.9659012**	**-75.5859598**
Cl^- K_h	**-454.4804216**	**-457.3535854**
$CuCl^0$ $C_{\infty v}$ (CRC)	**-2074.9596711**	**-2088.0092234**
$CuCl^0$ $C_{\infty v}$ (DM)	**-2075.0779378**	**-2088.3066213**
$CuCl^0$ triplet $C_{\infty v}$	**-2075.1549813**	**-2088.3107127**
$CuCl(H_2O)_1^0$ C_{2v} (CC)	**-2149.3327462**	**-2163.8807353**
$CuCl(H_2O)_1^0$ C_{2v} (DM)	[1+1]	-2163.9260017
$CuCl(H_2O)_1^0$ C_{2v} trip (DM)	-2150.0097685	-2163.9121895
$CuCl(H_2O)_1^0$ C_s #1 (CC)	-2149.9620149	-2163.6372335
$CuCl(H_2O)_1^0$ C_s #1 (DM)	-2150.0984411	-2163.9475744
$CuCl(H_2O)_1^0$ C_s #1 trip (DM)	**-2150.0571790**	-2163.9413146
$CuCl(H_2O)_1^0$ C_s #2 (CC)	**-2149.9762502**	**-2163.6451280**
$CuCl(H_2O)_1^0$ C_s #2 (DM)	-2150.1073401	**-2163.9561076**
$CuCl(H_2O)_1^0$ C_s #2 (DM)	**-2150.1702138**	
$CuCl(H_2O)_2^0$ C_{2v} #1 (CC)	-2224.9392078	-2239.2452603
$CuCl(H_2O)_2^0$ C_{2v} #1 (DM)	-2225.0787269	-2239.5933822
$CuCl(H_2O)_2^0$ C_{2v} #1 trip (DM)	-2225.1401732	-2239.5773416
$CuCl(H_2O)_2^0$ C_{2v} #2 (CC)	-2224.9326054	-2239.2329630
$CuCl(H_2O)_2^0$ C_{2v} #2 (DM)	-2225.0721849	-2239.5716874
$CuCl(H_2O)_2^0$ C_{2v} #2 trip (DM)	-2225.1342712	-2239.5606327
$CuCl(H_2O)_2^0$ C_s #1 (CC)	**-2224.9939135**	-2239.2952096
$CuCl(H_2O)_2^0$ C_s #1 (DM)	-2225.0735848	**-2239.5972696**
$CuCl(H_2O)_2^0$ C_s #1 trip (DM)	-2225.1839426	**-2239.5951549**
$CuCl(H_2O)_3^0$ C_{3v} #1 (CC)	(Cl^-) -2300.0051438	-2315.1306349
$CuCl(H_2O)_3^0$ C_{3v} #1 (DM)	(Cl^-) -2300.1285415	(Cl^-) **-2315.2289524**<br>(Cl^-) -2315.2352292
$CuCl(H_2O)_3^0$ C_{3v} #1 trip (DM)	(Cl^-) -2300.1926661	(Cl^-) **-2315.2379105**
$CuCl(H_2O)_3^0$ C_3 #1 (CC)	(Cl^-) **-2300.0053568**	(Cl^-) **-2314.9138112**
$CuCl(H_2O)_3^0$ C_3 #1 (DM)	(Cl^-) -2300.1285846	C_{3v} #1
$CuCl(H_2O)_3^0$ C_3 #1 trip (DM)	(Cl^-) -2299.9858083	(Cl^-) -2315.0194540
$CuCl(H_2O)_3^0$ C_s #1 (CC)		**-2315.1380990**
$CuCl(H_2O)_3^0$ C_1 #2 (DM)	(Cl^-) **-2300.0988322**	C_{3v} #1
$CuCl(H_2O)_3^0$ C_1 #2 trip (DM)	(Cl^-) **-2300.0429307**	C_{3v} #1

	HF/STO-3G	HF/3-21G
$CuCl(H_2O)_4^0$ C_{4v} #1 (CC)	(Cl^-) -2374.9631463	(Cl^-) -2390.5023028<br>(Cl^-) -2390.7176086
$CuCl(H_2O)_4^0$ C_{4v} #1 (DM)	(Cl^-) -2375.0883402	(Cl^-) -2390.8518739
$CuCl(H_2O)_4^0$ C_{4v} #1 trip (DM)	(Cl^-) -2374.9146905	(Cl^-) -2390.8529121
$CuCl(H_2O)_4^0$ C_4 #1 (CC)	(Cl^-) -2374.9774937	(Cl^-) -2390.5114156
$CuCl(H_2O)_4^0$ C_4 #1 (DM)	(Cl^-) -2375.1043018	(Cl^-) **-2390.8557287**
$CuCl(H_2O)_4^0$ C_4 #1 trip (DM)	(Cl^-) -2374.9403906	(Cl^-) **-2390.8569065**
$CuCl(H_2O)_4^0$ C_4 #2 [1+4] (CC)		-2390.4511135
$CuCl(H_2O)_4^0$ C_4 #2 [1+4] (DM)		-2390.7568133
$CuCl(H_2O)_4^0$ C_4 #2 trip [1+4] (DM)		-2390.7613346
$CuCl(H_2O)_4^0$ C_2 #1 (CC)	(Cl^-) -2374.9823844	(Cl^-) -2390.5385074
$CuCl(H_2O)_4^0$ C_2 #1 (DM)	(Cl^-) -2375.1068527	(Cl^-) -2390.8479079
$CuCl(H_2O)_4^0$ C_2 #1 trip (DM)	(Cl^-) -2374.9936255	C_4 #1
$CuCl(H_2O)_4^0$ [3+2] C_2 #2 (CC)		**-2390.7848069**
$CuCl(H_2O)_4^0$ [3+2] C_2 #2 (DM)		-2390.8449890
$CuCl(H_2O)_4^0$ [3+2] triplet C_2 #2 (DM)		-2390.8346997
$CuCl(H_2O)_5^0$ [5+1] C_{2v} #1 (CC)	-2449.8947927	
$CuCl(H_2O)_5^0$ [3+2+Cl^-] C_{2v} #2 (CC)		-2466.3641780
$CuCl(H_2O)_5^0$ [3+2+Cl^-] C_{2v} #2 (DM)		-2466.4145271
$CuCl(H_2O)_5^0$ [3+2+Cl^-] trip C_{2v} #2 (DM)		-2466.4155364
$CuCl(H_2O)_5^0$ [3+2+Cl^-] C_2 #1 (CC)		-2466.3807999
$CuCl(H_2O)_5^0$ [3+2+Cl^-] C_2 #1 (DM)		-2466.4329832
$CuCl(H_2O)_5^0$ [3+2+Cl^-] C_2 #1 (DM)		-2466.4340705
$CuCl(H_2O)_5^0$ [3+2+Cl^-] C_1 #1 (CC)	**-2450.0031232**	**-2466.3880127**
$CuCl(H_2O)_5^0$ [3+2+Cl^-] C_1 #1 (DM)	**-2450.1258663**	**-2466.4787587**
$CuCl(H_2O)_5^0$ [3+2+Cl^-] C_1 #1 (DM) triplet	**-2450.0284809**	**-2466.4798624**

	HF/STO-3G	HF/3-21G
$CuCl_2^-$ $D_{\infty h}$ (CC)	-2529.4684744	**-2545.6768739**
$CuCl_2^-$ $D_{\infty h}$ (DM)	-2529.6052618	-2545.7298892
$CuCl_2^-$ $D_{\infty h}$ trip (DM)	-2529.6680653	-2545.7036746
$CuCl_2(H_2O)^-$ C_{2v} #1	-2604.5270286	-2621.2808882
$CuCl_2(H_2O)^-$ C_{2v} #1 (DM)	-2604.6475643	-2621.3726070
$CuCl_2(H_2O)^-$ C_s #1	**-2604.5703961**	-2621.0623774
$CuCl_2(H_2O)^-$ C_s #1 (DM)	**-2604.7069939**	**-2621.3840398**
$CuCl_2(H_2O)_2^-$ C_{2v} #1 (CC)	**-2679.5600932**	**-2696.9270310**
$CuCl_2(H_2O)_2^-$ C_{2v} #1 (DM)	**-2679.6830356**	-2697.0126438
$CuCl_2(H_2O)_2^-$ C_{2v} #2 (CC)	-2679.5336499	-2696.6836539
$CuCl_2(H_2O)_2^-$ C_{2v} #2 (DM)	-2679.6583911	-2696.9799895
$CuCl_2(H_2O)_3^-$ D_{3h} #1 (CC)	-2754.3518270	($2Cl^-$) -2772.5009728
$CuCl_2(H_2O)_3^-$ D_{3h} #1 (DM)		2772.5773088
$CuCl_2(H_2O)_3^-$ D_{3h} #3 (CC)	-2754.3879151	-2772.4900353
$CuCl_2(H_2O)_3^-$ D_{3h} #3 (DM)	-2754.5387145	-2772.5794711
$CuCl_2(H_2O)_3^-$ C_{2v} [4+1] (CC)	-2754.5368360	-2772.5352636
$CuCl_2(H_2O)_3^-$ C_{2v} [4+1] (DM)	**-2754.6647110**	-2772.6201093
$CuCl_2(H_2O)_3^-$ C_s #1 [4+1] (CC)	-2754.5370806	**-2772.5318514**
$CuCl_2(H_2O)_3^-$ C_s #1 [4+1] (DM)	-2754.6621887	**-2772.6222983**
$CuCl_2(H_2O)_3^-$ C_s #2 [4+1] (CC)		-2772.5389296
$CuCl_2(H_2O)_3^-$ C_s #2 [4+1] (DM)		-2772.6182843
$CuCl_2(H_2O)_3^-$ C_s #3 [3+2] (CC)	-2754.5695305	-2772.5372359
$CuCl_2(H_2O)_3^-$ C_s #3 [3+2] (DM)	**-2754.6948354**	-2772.6255885
$CuCl_2(H_2O)_3^-$ C_1 #1 [3+2] (CC)	**-2754.5629625**	**-2772.7361438**
$CuCl_2(H_2O)_3^-$ C_1 #1 [3+2] (DM)	**-2754.6852777**	**-2772.6477492**
$CuCl_2(H_2O)_3^-$ C_1 #2 [3+2] (CC)	**-2754.5952353**	**-2772.7451441**
$CuCl_2(H_2O)_3^-$ C_1 #2 [3+2] (DM)	**-2754.7173003**	**-2772.6497953**
$CuCl_2(H_2O)_4^-$ D_{4h} #1 (CC)	-2829.3106585	($2Cl^-$) -2848.0586417
$CuCl_2(H_2O)_4^-$ D_{4h} #1 (DM)	waters dissociated	-2848.1575438
$CuCl_2(H_2O)_4^-$ D_{4h} #2 [2+4] (CC)	-2829.3596810	-2848.0807380
$CuCl_2(H_2O)_4^-$ D_{4h} #2 [2+4] (DM)	-2829.4972856	-2848.1143650
$CuCl_2(H_2O)_4^-$ C_{2v} #1 [2+4] (CC)	-2829.4891523	
$CuCl_2(H_2O)_4^-$ C_{2v} #1 [2+4] (DM)	-2829.6112777	-2848.2067805
$CuCl_2(H_2O)_4^-$ C_{2v} #2 [5+1] (CC)		-2848.1276486
$CuCl_2(H_2O)_4^-$ C_{2v} #2 [5+1] (DM)		-2848.2137827
$CuCl_2(H_2O)_4^-$ C_{2v} #3 [4+2] (CC)	**-2829.5185082**	-2847.9034162
$CuCl_2(H_2O)_4^-$ C_{2v} #3 [4+2] (DM)		-2848.2198586
$CuCl_2(H_2O)_4^-$ C_s #1 [4+2] (CC)	-2829.5212289	-2848.1460219
$CuCl_2(H_2O)_4^-$ C_s #1 [4+2] (DM)	-2829.6460516	-2848.2235796
$CuCl_2(H_2O)_4^-$ C_2 #1 [4+2Cl^-] (CC)	-2829.5186348	-2848.1912599
$CuCl_2(H_2O)_4^-$ C_2 #1 [4+2Cl^-] (DM)	-2829.6437070	**-2848.2908238**
$CuCl_2(H_2O)_4^-$ C_1 #1 [2+3+Cl^-] (CC)	**-2829.5722513**	**-2848.1771553**
$CuCl_2(H_2O)_4^-$ C_1 #1 [2+3+Cl^-] (DM)	**-2829.6940884**	**-2848.2566014**
$CuCl_2(H_2O)_4^-$ C_1 #2 [2+3+Cl^-] (DM)		**-2848.2455004**

	HF/STO-3G	HF/3-21G
$CuCl_3^{2-}$ D_{3h} (CC)	-2983.8736544	**-3002.9456898**
$CuCl_3^{2-}$ C_{3v} (CC)	**-2984.0140002**	
$CuCl_3(H_2O)^{2-}$ C_{2v} #1 [3+1]		**-3078.5662179**
$CuCl_3(H_2O)^{2-}$ C_s #1	C_s #1 [3+1]	
$CuCl_3(H_2O)^{2-}$ C_s #2	C_s #2 [3+1]	
$CuCl_3(H_2O)^{2-}$ C_s #1 [3+1]	**-3059.0047535**	-3078.5592064
$CuCl_3(H_2O)^{2-}$ C_s #2 [3+1]	-3059.0017821	
$CuCl_3(H_2O)_2^{2-}$ C_{2v} #1 [3+2]	-3133.9101029	**-3154.1833543**
$CuCl_3(H_2O)_2^{2-}$ C_{2v} #2 [3+2]	**-3133.9933979**	**-3154.1847700**
$CuCl_3(H_2O)_2^{2-}$ C_s #1 [3+2]	-3133.9932699	-3154.1842658
$CuCl_3(H_2O)_2^{2-}$ C_s #4 [4+Cl^-]	-3133.9801720	-3154.1959655
$CuCl_3(H_2O)_3^{2-}$ D_{3h} [3+3]	-3208.8478801	**-3229.8013750**
$CuCl_3(H_2O)_3^{2-}$ C_{3v} [3+3]	**-3208.9900035**	D_{3h}
$CuCl_3(H_2O)_3^{2-}$ C_{2v} #2 [3+3Cl^-]		-3229.8656563
$CuCl_3(H_2O)_3^{2-}$ C_{2v} #2 [3+3Cl^-]		-3229.8048460
$CuCl_3(H_2O)_3^{2-}$ C_s [3+3]	-3209.0193051	-3229.8697990
$CuCl_3(H_2O)_3^{2-}$ C_2 [3+3]		-3229.8721694
$CuCl_3(H_2O)_3^{2-}$ C_1 [3+1+2Cl^-]	**-3209.0395734**	**-3229.8817642**
$CuCl_4^{3-}$ T_d	**-3437.6499363**	-3460.0740946

	HF/6-31G*	HF/6-31+G*	HF/6-311+G*
Cu^+ K_h	(d^{10}) **-1638.3528406**<br>**-1638.0417719**	(d^{10}) **-1638.4550683**<br>**-1638.0539765**	**-1638.6050297**
H_2O C_{2v}	**-76.0107465**	**-76.0177432**	**-76.0377596**
Cl^- K_h	**-459.5259969**	**-459.5396601**	**-459.5654249**
$CuCl^0$ $C_{\infty v}$ (CC)	**-2097.8054824**	**-2097.8268954**	
$CuCl^0$ $C_{\infty v}$	**-2098.1407917**	**-2098.2405058**	**-2098.4136254**
$CuCl^0$ $C_{\infty v}$ (DM)	**-2098.1747677**	**-2098.2177318**	
$CuCl^0$ triplet $C_{\infty v}$	**-2098.1788759**	**-2098.2215554**	
$CuCl(H_2O)_1^0$ C_{2v}	-2174.2025034	-2174.2897129	**-2174.4847438**
$CuCl(H_2O)_1^0$ C_{2v} (DM)	-2174.2032581		
$CuCl(H_2O)_1^0$ C_{2v} trip (DM)	-2174.1914015		
$CuCl(H_2O)_1^0$ C_s #1 (CC)	-2173.8535269		
$CuCl(H_2O)_1^0$ C_s #1 (DM)	-2174.2083089		
$CuCl(H_2O)_1^0$ C_s #1 trip (DM)	-2174.2145875		
$CuCl(H_2O)_1^0$ C_s #2 (CC)	**-2173.8561866**		
$CuCl(H_2O)_1^0$ C_s #2 (DM)	-2174.2032752		
$CuCl(H_2O)_1^0$ C_s #2 (DM)	-2174.2181914		
$CuCl(H_2O)_1^0$ C_s #3	**-2174.2025222**	**-2174.2897174**	n/a
$CuCl(H_2O)_1^0$ C_1 #1 trip (DM)	**-2174.2183101**	**-2174.2588276**	
$CuCl(H_2O)_2^0$ C_{2v} #1 (CC)	-2249.8541206		
$CuCl(H_2O)_2^0$ C_{2v} #1 (DM)	-2250.2431370		
$CuCl(H_2O)_2^0$ C_{2v} #1 trip (DM)	-2250.2405612		
$CuCl(H_2O)_2^0$ C_{2v} #2 (CC)	-2249.8497616		
$CuCl(H_2O)_2^0$ C_{2v} #2 (DM)	-2250.2336539		
$CuCl(H_2O)_2^0$ C_{2v} #2 trip (DM)	-2250.2341422		
$CuCl(H_2O)_2^0$ C_{2v} #3	-2250.2285239	-2250.3179173	-2250.5335056
$CuCl(H_2O)_2^0$ C_{2v} #4	-2250.2309373	-2250.3183883	-2250.5353833
$CuCl(H_2O)_2^0$ C_2 #1	-2250.2310961	-2250.3200032	-2250.5359711
$CuCl(H_2O)_2^0$ C_s #1 (CC)	**-2249.9184555**		
$CuCl(H_2O)_2^0$ C_s #1 (DM)	**-2250.2450394**	**-2250.2891672**	
$CuCl(H_2O)_2^0$ C_s #1 trip (DM)	**-2250.2479431**	**-2250.2894609**	
$CuCl(H_2O)_2^0$ C_s #2	C_{2v} #4	-2250.3185254	
$CuCl(H_2O)_2^0$ C_s #3	-2250.2309457	[2+1]	-2250.5360641
$CuCl(H_2O)_2^0$ C_s #4 [2+1]	-2250.2336903	-2250.3242961	**-2250.5411008**
$CuCl(H_2O)_2^0$ C_s #5 [2+1]	-2250.2318753	-2250.3242454	-2250.5409300
$CuCl(H_2O)_2^0$ C_s #6	-2250.2290687	-2250.3185474	-2250.5343862
$CuCl(H_2O)_2^0$ [2+1] C_1 #1	**-2250.2348946**	**-2250.3243758**	C_s #4
$CuCl(H_2O)_2^0$ [2+1] C_1 #2	C_1 #1	**-2250.3250786**	**-2250.5411187**

	HF/6-31G*	HF/6-31+G*	HF/6-311+G*
$CuCl(H_2O)_3^0$ C_{3v} #1	-2326.2627195	-2326.3486034	-2326.5876190
$CuCl(H_2O)_3^0$ C_{3v} #2	-2326.2550921	-2326.3455867	-2326.5817399
$CuCl(H_2O)_3^0$ C_3 #1 (CC)	(Cl^-) **-2325.8988759**	(Cl^-) **-2325.9224050**	
$CuCl(H_2O)_3^0$ C_3 #1 (DM)	(Cl^-) -2326.2707667	**-2326.3091181**	
$CuCl(H_2O)_3^0$ C_3 #1 trip (DM)	(Cl^-) -2326.1024328 -2326.2647121	**-2326.3147407**	
$CuCl(H_2O)_3^0$ C_3 #2	**-2326.2652879**	**-2326.3521078**	**-2326.5893235**
$CuCl(H_2O)_3^0$ C_3 #2 (DM)	-2326.2646160		
$CuCl(H_2O)_3^0$ C_3 #2 trip (DM)	**-2326.2662788**		
$CuCl(H_2O)_3^0$ C_s #1	-2326.2636445	-2326.3499733	-2326.5879705
$CuCl(H_2O)_3^0$ C_s #2 [2+2]	-2326.2641231 [4+0]	**-2326.3566167**	**-2326.5933211**
$CuCl(H_2O)_3^0$ C_1 #2 (DM)	C_3 #1		
$CuCl(H_2O)_3^0$ C_1 #2 trip (DM)	C_3 #1		
$CuCl(H_2O)_3^0$ [2+2] C_1 #3	**-2326.2691053**	**-2326.3649971**	**-2326.6028408**

	HF/6-31G*	HF/6-31+G*	HF/6-311+G*
$CuCl(H_2O)_4^0$ C_{4v} #1	(Cl^-) -2402.2800674	-2402.3724001	-2402.6318245
$CuCl(H_2O)_4^0$ C_{4v} #1 (DM)	(Cl^-) -2402.0813463<br>(Cl^-) -2402.2864186		
$CuCl(H_2O)_4^0$ C_{4v} #1 trip (DM)	(Cl^-) -2402.2887341		
$CuCl(H_2O)_4^0$ C_{4v} #2	-2402.2644466	-2402.3654256	-2402.6219720
$CuCl(H_2O)_4^0$ C_4 #1 (CC)	(Cl^-) -2401.9166463<br>-2402.2898564		
$CuCl(H_2O)_4^0$ C_4 #1 (DM)	(Cl^-) **-2402.3004195**		
$CuCl(H_2O)_4^0$ C_4 #1 trip (DM)	(Cl^-) -2402.2961170		
$CuCl(H_2O)_4^0$ C_4 #1	-2402.2901960	-2402.3827353	-2402.6401482
$CuCl(H_2O)_4^0$ C_4 #2 [1+4] (CC)	-2402.2506252		
$CuCl(H_2O)_4^0$ C_4 #2 [1+4]	-2402.2872828	C_4 #1	C_4 #1
$CuCl(H_2O)_4^0$ C_4 #2 trip [1+4] (DM)	-2402.2693760		
$CuCl(H_2O)_4^0$ C_{2v} #1	-2402.2823583	-2402.3752570	-2402.6329650
$CuCl(H_2O)_4^0$ C_{2v} #2	n/a	-2402.3741976	n/a
$CuCl(H_2O)_4^0$ C_{2v} #3	C_{2v} #1	C_{2v} #1	C_{2v} #1
$CuCl(H_2O)_4^0$ C_{2v} #4	-2402.2807412	-2402.3749799	-2402.6329290
$CuCl(H_2O)_4^0$ C_{2v} #5	-2402.2873152	-2402.3809034	-2402.6382793
$CuCl(H_2O)_4^0$ C_2 #1 (CC)	(Cl^-) **-2402.2902691**	C_2 #2	C_2 #2
$CuCl(H_2O)_4^0$ C_2 #1 (DM)	C_4 #1		
$CuCl(H_2O)_4^0$ C_2 #1 trip (DM)	C_4 #1 **-2402.3028845**		
$CuCl(H_2O)_4^0$ [3+2] C_2 #2	**-2402.3035922**	**-2402.3993223**	**-2402.6588955**
$CuCl(H_2O)_4^0$ [3+2] C_2 #2 (DM)	**-2402.3036110**	-2402.3525885	
$CuCl(H_2O)_4^0$ [3+2] triplet C_2 #2 (DM)	-2402.3014121		
$CuCl(H_2O)_4^0$ [3+2] C_2 #4	C_2 #2		
$CuCl(H_2O)_4^0$ [4+1] C_s #1	-2402.2973074	-2402.3902404	-2402.6489689
$CuCl(H_2O)_4^0$ [4+1] C_s #2	C_s #1	-2402.3905164	-2402.6491418
$CuCl(H_2O)_4^0$ [4+1] C_s #3	-2402.2848464	-2402.3779313	-2402.6353390
$CuCl(H_2O)_4^0$ [3+2] C_s #4	-2402.2936114	-2402.3909778	-2402.6493786
$CuCl(H_2O)_4^0$ [4+1] C_s #5	**-2402.3034408**	**-2402.3988809**	**-2402.6585969**
$CuCl(H_2O)_4^0$ [5+0] C_s #6	-2402.2932512	C_s #4	C_s #4
$CuCl(H_2O)_4^0$ [2+3] C_s #7	**-2402.2974548**	**-2402.3971593**	**-2402.6554250**
$CuCl(H_2O)_4^0$ [3+2] C_1 #3	**-2402.2980944**	C_1 #4	C_1 #4
$CuCl(H_2O)_4^0$ [2+2+1] C_1 #4	**-2402.2973319**	**-2402.3991819**	**-2402.6592307**
$CuCl(H_2O)_4^0$ [2+3] C_1 #5	C_1 #4	C_1 #4	C_1 #4
$CuCl(H_2O)_4^0$ C_1 #6 [4+1]	**-2402.2990594**	**-2402.3908224**	**-2402.6491505**

$CuCl(H_2O)_4^0$ *C_1* #7 [3+1+Cl^-]	*C_1* #6	*C_1* #6	*C_1* #6
$CuCl(H_2O)_4^0$ *C_1* #8 [3+2]	**-2402.2983801**	**-2402.3946729**	**-2402.6530280**
$CuCl(H_2O)_4^0$ *C_1* #9 [4+1]	**-2402.2989836**<br>**-2402.3000082**	**-2402.3906153**	**-2402.6494579**
$CuCl(H_2O)_4^0$ *C_1* #10 [3+2]	**-2402.2993046**	**-2402.3964606**	**-2402.6560182**
$CuCl(H_2O)_4^0$ *C_1* #11 [3+2]		**-2402.3921730**	

	HF/6-31G*	HF/6-31+G*	HF/6-311+G*
$CuCl(H_2O)_5{}^0$ [5+Cl⁻] C_{2v} #1	-2478.3127183	-2478.4048554	-2478.6832479
$CuCl(H_2O)_5{}^0$ [5+Cl⁻] C_{2v} #2	-2478.3108501	-2478.4020963	-2478.6811316
$CuCl(H_2O)_5{}^0$ [5+Cl⁻] C_{2v} #3	-2478.3085459	-2478.4029332	-2478.6803650
$CuCl(H_2O)_5{}^0$ [2+4] C_{2v} #4	[4+2]-2478.2962309	-2478.4080695	-2478.6871672
$CuCl(H_2O)_5{}^0$ [5+Cl⁻] C_{2v} #5	-2478.3155659	-2478.4048125	-2478.6854778
$CuCl(H_2O)_5{}^0$ [5+Cl⁻] C_{2v} #6	-2478.3173086		[4+2]-2478.6869418
$CuCl(H_2O)_5{}^0$ [3+2+Cl⁻] C_{2v} #2 (CC,DM)	-2478.3034215		
$CuCl(H_2O)_5{}^0$ [3+2+Cl⁻] trip C_{2v} #2 (DM)	C_{2v} #4		
$CuCl(H_2O)_5{}^0$ [4+2] C_{2v} #3 (CC)	-2478.2991715		
$CuCl(H_2O)_5{}^0$ [4+2] C_{2v} #3 (DM)	-2478.2934171		
$CuCl(H_2O)_5{}^0$ [2+3+Cl⁻] trip C_{2v} #4 (DM)	-2478.2833439		
$CuCl(H_2O)_5{}^0$ [3+3] trip C_{2v} #5 (DM)	-2478.2756144		
$CuCl(H_2O)_5{}^0$ [4+2] C_2 #2 (CC)	-2478.3204641	-2478.4248668	
$CuCl(H_2O)_5{}^0$ [5+ Cl⁻] C_2#3 (CC)	**-2478.3213334**		
$CuCl(H_2O)_5{}^0$ [4+ 1+Cl⁻] C_2#5 (DM)	-2478.3111556		
$CuCl(H_2O)_5{}^0$ [4+ 1+Cl⁻] C_2#5 (DM)	-2478.3136302		
$CuCl(H_2O)_5{}^0$ [3+3] C_s #1 (DM) triplet	**-2478.3419227**	**-2478.3920280**	
$CuCl(H_2O)_5{}^0$ [3+2+Cl⁻] C_1 #1 (CC)		**-2478.4299082**	
$CuCl(H_2O)_5{}^0$ [4+2] C_1 #1 (CC)	**-2478.3299471**		
$CuCl(H_2O)_5{}^0$ [2+4] C_{2v} #1	-2478.3192892	-2478.4262366	-2478.7074437
$CuCl(H_2O)_5{}^0$ [2+4] C_2 #1	-2478.3232818	-2478.4278564	-2478.7090584
$CuCl(H_2O)_5{}^0$ [2+4] C_s #1	**-2478.3263600**	**-2478.4321911**	**-2478.7122531**
$CuCl(H_2O)_7{}^0$ [2+4] C_{2v} #1	-2630.3734015	-2630.4946704	-2630.8203244
$CuCl(H_2O)_7{}^0$ [2+4] C_2 #1	-2630.3813200	-2630.4989290	-2630.8241848
$CuCl(H_2O)_7{}^0$ [2+4] C_s #1	**-2630.3825872**	**-2630.5002516**	**-2630.8248818**
Cu_3Cl_3 D_{3h}	**-6294.6151464**	**-6294.8581591**	**-6295.3759738**

	HF/6-31G*	HF/6-31+G*	HF/6-311+G*
$CuCl_2^-$ $D_{\infty h}$	**-2557.7771086**	**-2557.8731890**	**-2558.0710650**
$CuCl_2^-$ $D_{\infty h}$ (DM)	**-2557.7771086**	**-2557.8731890**	
$CuCl_2^-$ $D_{\infty h}$ trip (DM)	-2557.7478147	-2557.7976212	
$CuCl_2(H_2O)^-$ C_{2v} #1	-2633.7881482	-2633.8874892	-2634.1070522
$CuCl_2(H_2O)^-$ C_{2v} #1 (DM)	-2633.7881482		
$CuCl_2(H_2O)^-$ C_{2v} #2	-2633.7814781	water dissociates	-2634.1008829
$CuCl_2(H_2O)^-$ C_{2v} #3 [2+1]	**-2633.8032905**	-2633.9032484	-2634.1212416
$CuCl_2(H_2O)^-$ C_{2v} #3 [2+1] (DM)	**-2633.8032903**		
$CuCl_2(H_2O)^-$ C_s #1	C_{2v} #3 [2+1]	C_{2v} #3 [2+1]	C_{2v} #3 [2+1]
$CuCl_2(H_2O)^-$ C_s #2	C_s #3 [2+1]	C_s #3 [2+1]	C_s #3 [2+1]
$CuCl_2(H_2O)^-$ C_s #3 [2+1]	**-2633.8018639**	**-2633.9039656**	**-2634.1223099**
$CuCl_2(H_2O)^-$ C_s #4 [2+1]	-2633.8014678	-2633.9037377	**-2634.1221786**
$CuCl_2(H_2O)_2^-$ D_{2h} #1	-2709.7963606	-2709.9004864	-2710.1424507
$CuCl_2(H_2O)_2^-$ D_{2h} #2	water dissociates	water dissociates	water dissociates
$CuCl_2(H_2O)_2^-$ D_{2h} #3 [2+2]	-2709.8256003	-2709.9308448	-2710.1688970
$CuCl_2(H_2O)_2^-$ C_{2h} #1	D_{2h} #3 [2+2]	D_{2h} #3 [2+2]	D_{2h} #3 [2+2]
$CuCl_2(H_2O)_2^-$ C_{2h} #2	C_{2h} #3 [2+2]	C_{2h} #3 [2+2]	C_{2h} #3 [2+2]
$CuCl_2(H_2O)_2^-$ C_{2h} #3 [2+2]	-2709.8258905	-2709.9341194	-2710.1729411
$CuCl_2(H_2O)_2^-$ C_{2v} #1 [3+Cl^-]	-2709.8268844	water dissociates	-2710.1644054
$CuCl_2(H_2O)_2^-$ C_{2v} #2	water dissociates	water dissociates	water dissociates
$CuCl_2(H_2O)_2^-$ C_{2v} #2 (CC)	-2709.4436245		
$CuCl_2(H_2O)_2^-$ C_{2v} #2 (DM)	-2709.7976476		
$CuCl_2(H_2O)_2^-$ C_{2v} #3 [2+2]	-2709.8257228	-2709.9339691	-2710.1728067
$CuCl_2(H_2O)_2^-$ C_{2v} #4 [2+2]	-2709.8252159	**-2709.9335332**	**-2710.1724895**
$CuCl_2(H_2O)_2^-$ C_{2v} #5 [2+2]	**-2709.8282805**	-2709.9320336	-2710.1703079
$CuCl_2(H_2O)_2^-$ C_{2v} #5 [2+2] (DM)	**-2709.8282805**		
$CuCl_2(H_2O)_2^-$ C_2 #1 [2+2]	**-2709.8259652**	**-2709.9341225**	**-2710.1729434**
$CuCl_2(H_2O)_2^-$ C_2 #2 [2+2]	**-2709.8260749**	C_2 #1 [2+2]	**-2710.1730327**
$CuCl_2(H_2O)_2^-$ C_2 #3 [2+2]	-2709.8252224	n/a	n/a
$CuCl_2(H_2O)_2^-$ C_2 #4 [2+2]	n/a	C_2 #1 [2+2]	C_2 #1 [2+2]
$CuCl_2(H_2O)_2^-$ C_i #1 [2+2]	-2709.8259774	n/a	-2710.1729405
$CuCl_2(H_2O)_2^-$ C_s #1 [2+2]	n/a	C_{2v} #4 [2+2]	C_{2v} #4 [2+2]
$CuCl_2(H_2O)_2^-$ C_s #2 [2+2]	-2709.8258647	-2709.9340568	-2710.1729493
$CuCl_2(H_2O)_2^-$ C_s #3 [2+2]	-2709.8252248	n/a	n/a
$CuCl_2(H_2O)_2^-$ C_s #4 [2+2]	n/a	n/a	n/a
$CuCl_2(H_2O)_2^-$ C_1 #1 [2+2]	**-2709.8313593**	**-2709.9370764**	**-2710.1760281**

	HF/6-31G*	HF/6-31+G*	HF/6-311+G*
$CuCl_2(H_2O)_3^-$ D_{3h} #1	-2785.8159932	-2785.9164956	-2786.1817545
$CuCl_2(H_2O)_3^-$ D_{3h} #1 (DM)	-2785.8159931		
$CuCl_2(H_2O)_3^-$ D_{3h} #2	-2785.7834088	waters dissociated	waters dissociated
$CuCl_2(H_2O)_3^-$ D_{3h} #3 [2+3]	**-2785.8516569**	-2785.9582097	-2786.2162743
$CuCl_2(H_2O)_3^-$ D_{3h} #3 [2+3] (DM)	**-2785.8516569**		
$CuCl_2(H_2O)_3^-$ C_{3h} #1	D_{3h} #3 [2+3]	D_{3h} #3 [2+3]	D_{3h} #3 [2+3]
$CuCl_2(H_2O)_3^-$ C_{3v} #1 [4+Cl^-]	-2785.8485355	-2785.9472698	-2786.2106687
$CuCl_2(H_2O)_3^-$ C_{3v} #2 [2+3]	-2785.8468702	-2785.9615096	-2786.2207975
$CuCl_2(H_2O)_3^-$ C_{2v} #1 [3+2]	-2785.8464329	-2785.9516897	-2786.2123719
$CuCl_2(H_2O)_3^-$ C_{2v} #2 [3+2 Cl^-]	C_{2v} #1 [3+2]	C_{2v} #3 [2+3]	C_{2v} #3 [2+3]
$CuCl_2(H_2O)_3^-$ C_{2v} #3 [2+3]	n/a	-2785.9582099	-2786.2163412
$CuCl_2(H_2O)_3^-$ C_{2v} #4 [2+3]	-2785.8488868	-2785.9585831	-2786.2171093
$CuCl_2(H_2O)_3^-$ C_{2v} [4+1] (CC)	-2785.8434288		
$CuCl_2(H_2O)_3^-$ C_{2v} [4+1] (DM)	-2785.8587173		
$CuCl_2(H_2O)_3^-$ C_3 #1 [4+Cl^-]	-2785.8538796	**-2785.9541353**	**-2786.2155552**
$CuCl_2(H_2O)_3^-$ C_3 #2 [2+3]	-2785.8470020	-2785.9615367	-2786.2209110
$CuCl_2(H_2O)_3^-$ C_2 #1 [3+2 Cl^-]	-2785.8434008	-2785.9354237	-2786.2001601
$CuCl_2(H_2O)_3^-$ C_2 #2 [3+2]	-2785.8560385	-2785.9633021	-2786.2247610
$CuCl_2(H_2O)_3^-$ C_2 #3 [3+2]	-2785.8443941	-2785.9527997	-2786.2133762
$CuCl_2(H_2O)_3^-$ C_2 #5 [2+3]	**-2785.8573342**	-2785.9662647	**-2786.2255227**
$CuCl_2(H_2O)_3^-$ C_2 #6 [2+3]	C_2 #5 [2+3]	-2785.9626135	-2786.2216029
$CuCl_2(H_2O)_3^-$ C_2 #7 [2+3]	C_2 #5 [2+3]	C_2 #6 [2+3]	C_2 #6 [2+3]
$CuCl_2(H_2O)_3^-$ C_2 #8 [2+3]	n/a	C_2 #5 [2+3]	C_2 #6 [2+3]
$CuCl_2(H_2O)_3^-$ C_s #1 [4+1] (CC)	-2785.8526292		
$CuCl_2(H_2O)_3^-$ C_s #1 [4+1] (DM)	-2785.8526290		
$CuCl_2(H_2O)_3^-$ C_s #2 [4+1] (CC)	C_1 #1 [4+ Cl^-]		
$CuCl_2(H_2O)_3^-$ C_s #2 [4+1] (DM)	-2785.8501920		
$CuCl_2(H_2O)_3^-$ C_s #3 [4+1] (CC)	-2785.8513143		
$CuCl_2(H_2O)_3^-$ C_s #3 [4+1] (DM)	-2785.8533003		
$CuCl_2(H_2O)_3^-$ C_s #1 [4+ Cl^-]	-2785.8505039	-2785.9521958	-2786.2139737
$CuCl_2(H_2O)_3^-$ C_s #2 [3+1+Cl^-]	**-2785.8549859**	**-2785.9552886**	**-2786.2168423**
$CuCl_2(H_2O)_3^-$ C_s #3 [2+3]	-2785.8483940	-2785.9629564	-2786.2223657
$CuCl_2(H_2O)_3^-$ C_s #4 [2+3]	-2785.8487127	-2785.9630419	-2786.2224958
$CuCl_2(H_2O)_3^-$ C_s #5 [2+3]	-2785.8468703	-2785.9615096	-2786.2207976
$CuCl_2(H_2O)_3^-$ C_s #6 [2+3]	n/a	C_{2v} #4 [2+3]	C_{2v} #4 [2+3]
$CuCl_2(H_2O)_3^-$ C_s #7 [2+3]	n/a	-2785.9630982	C_s #4 [2+3]
$CuCl_2(H_2O)_3^-$ C_s #8 [2+3]	n/a	C_s #7 [2+3]	C_s #4 [2+3]
$CuCl_2(H_2O)_3^-$ C_s #9 [2+3]	n/a	C_{2v} #4 [2+3]	C_{2v} #4 [2+3]
$CuCl_2(H_2O)_3^-$ C_1 #1 [3+2] (CC)	**-2785.8559765**	**-2785.9557099**	
$CuCl_2(H_2O)_3^-$ C_1 #1 [3+2] (DM)	**-2785.8559765**		
$CuCl_2(H_2O)_3^-$ C_1 #2 [3+2] (CC)	**-2785.8600305**	**-2785.9570164**	
$CuCl_2(H_2O)_3^-$ C_1 #2 [3+2] (DM)	**-2785.8600305**		
$CuCl_2(H_2O)_3^-$ C_1 #1 [4+ Cl^-]	**-2785.8549708**	C_s #2 [3+1+Cl^-]	C_s #2 [3+1+Cl^-]
$CuCl_2(H_2O)_3^-$ C_1 #2 [3+1+Cl^-]	n/a	**-2785.9580572**	**-2786.2199442**

$CuCl_2(H_2O)_3^-$ C_1 #3 [2+2+Cl$^-$]	n/a	n/a	n/a
$CuCl_2(H_2O)_3^-$ C_1 #4 [3+2Cl$^-$]	C_1 #1 [4+ Cl$^-$]		**-2786.2009069**
$CuCl_2(H_2O)_3^-$ C_1 #5 [3+2]	**-2785.8573198**	**-2785.9649406**	**-2786.2254581**
$CuCl_2(H_2O)_3^-$ C_1 #6 [3+2]	**-2785.8572616**		**-2786.2325861**
$CuCl_2(H_2O)_3^-$ C_1 #7 [2+3]	**-2785.8541237**	n/a	n/a

	HF/6-31G*	HF/6-31+G*	HF/6-311+G*
$CuCl_2(H_2O)_4^-$ D_{4h} #1 (CC,DM)	-2861.8142995		
$CuCl_2(H_2O)_4^-$ D_{4h} #2 [2+4] (CC)	-2861.8679981	-2861.9837240	-2862.2622521
$CuCl_2(H_2O)_4^-$ C_{2v} #1 [2+4] (CC,DM)	-2861.8699752		
$CuCl_2(H_2O)_4^-$ C_{2v} #2 [5+1] (CC,DM)	-2861.8488601		
$CuCl_2(H_2O)_4^-$ C_{2v} #3 [4+2] (CC,DM)	-2861.8702956		
$CuCl_2(H_2O)_4^-$ C_s #1 [4+2] (CC,DM)	-2861.8831320		
$CuCl_2(H_2O)_4^-$ C_2 #1 [4+2Cl$^-$] (CC,DM)	**-2861.8877505**	**-2861.9818656**	
$CuCl_2(H_2O)_4^-$ C_1 #1 [2+3+Cl$^-$] (CC,DM)	**-2861.8881110**		
$CuCl_2(H_2O)_4^-$ C_1 #1 [2+3+Cl$^-$] (DM)	**-2861.8887535**		
$CuCl_2(H_2O)_6^-$ D_{3d} #1 [2+6]	-3013.9106160	-3014.0442387	-3014.3647933
$CuCl_2(H_2O)_6^-$ S_6 #1 [2+6]	-3013.9129707<br>-3013.9451965	-3014.0677468	-3014.3888861
$CuCl_2(H_2O)_6^-$ S_6 #2 [2+6]	-3013.9440110	**-3014.0635525**	**-3014.3857714**
$CuCl_2(H_2O)_6^-$ D_3 #1 [2+6]	-3013.9247032	-3014.0521953	-3014.3724377
$CuCl_2(H_2O)_6^-$ D_3 #2 [2+6]	**-3013.9451958**	-3014.0677460	**-3014.3887260**
$CuCl_2(H_2O)_6^-$ C_{3h} #1 [2+6]	-3013.9451960	**-3014.0677462**	-3014.3888858
$CuCl_2(H_2O)_6^-$ C_3 #1 [2+6]	-3013.9247959	S_6 #2 [2+6]	S_6 #2 [2+6]

	HF/6-31G*	HF/6-31+G*	HF/6-311+G*
$CuCl_3^{2-}$ D_{3h}	**-3017.2135372**	**-3017.3227657**	**-3017.5470817**
$CuCl_3(H_2O)^{2-}$ C_{2v} #1 [3+1]	**-3093.2551044**	**-3093.3690407**	**-3093.6140360**
$CuCl_3(H_2O)^{2-}$ C_s #1	C_s #1 [3+1]	C_s #1 [3+1]	C_s #1 [3+1]
$CuCl_3(H_2O)^{2-}$ C_s #2	[2+2Cl^-]	[2+2Cl^-]	[2+2Cl^-]
$CuCl_3(H_2O)^{2-}$ C_s #1 [3+1]	-3093.2480705	-3093.3623240	-3093.6072354
$CuCl_3(H_2O)_2^{2-}$ C_{2v} #1 [3+2]	**-3169.2927406**	**-3169.4110756**	**-3169.6767637**
$CuCl_3(H_2O)_2^{2-}$ C_{2v} #2 [3+2]	**-3169.2946630**	**-3169.4137289**	**-3169.6795145**
$CuCl_3(H_2O)_2^{2-}$ C_s #1 [3+2]	-3169.2816755		
$CuCl_3(H_2O)_2^{2-}$ C_s #4 [4+Cl^-]	-3169.2849154		
$CuCl_3(H_2O)_3^{2-}$ D_{3h} [3+3]	**-3245.3321857**	**-3245.4567350**	**-3245.7433713**
$CuCl_3(H_2O)_3^{2-}$ C_{2v} #2 [3+3Cl^-]	-3245.3207348		
$CuCl_3(H_2O)_3^{2-}$ C_s [3+3]	-3245.3318329		
$CuCl_3(H_2O)_3^{2-}$ C_2 [3+3]	-3245.3255317		
$CuCl_3(H_2O)_3^{2-}$ C_1 [3+1+2Cl^-]	**-3245.3347851**	**-3245.4446824**	
$CuCl_3(H_2O)_3^{2-}$ C_s #1 [3+3]	**-3245.3305271**	**-3245.4542564**	**-3245.7406947**
$CuCl_3(H_2O)_4^{2-}$ C_{2v} #1 [3+4]	**-3321.3662563**	**-3321.4956616**	**-3321.8028901**
$CuCl_3(H_2O)_4^{2-}$ C_{2v} #2 [3+4]	**-3321.3645334**	**-3321.4929702**	**-3321.8000545**
$CuCl_3(H_2O)_5^{2-}$ C_{2v} #1 [3+5]	**-3397.3985192**	**-3397.5327568**	**-3397.8605649**
$CuCl_3(H_2O)_6^{2-}$ D_{3h} #1 [3+6]	**-3473.4291221**	**-3473.5680891**	**-3473.9164896**
$CuCl_4^{3-}$ T_d (CC)	-3476.2026038		
$CuCl_4^{3-}$ T_d	-3476.5385381	-3476.6701109	-3476.9206517
$CuCl_4(H_2O)_6^{3-}$ T_d [4+6]	**-3932.8127846**	**-3932.9680263**	**-3933.3431365**

	B3LYP/6-31G*	B3LYP/6-31+G*	B3LYP/6-311+G*
Cu^+ K_h	**-1639.8608850**	**-1640.0169542**	**-1640.1769055**
H_2O C_{2v}	**-76.4089540**	**-76.4225732**	**-76.4438104**
Cl^- K_h	**-460.2522333**	**-460.2747257**	**-460.3037246**
$CuCl^0$ $C_{\infty v}$	**-2100.4513383**	**-2100.5824128**	**-2100.7667183**
$CuCl(H_2O)_1$ C_{2v}	-2176.9309364	-2177.0480711	-2177.2559624
$CuCl(H_2O)_1$ C_s #3	**-2176.9323862**	**-2177.0491199**	**-2177.2561835**
$CuCl(H_2O)_2^0$ C_{2v} #3	-2253.3554707	-2253.4769168	-2253.7058578
$CuCl(H_2O)_2^0$ C_{2v} #4	-2253.3552279	-2253.4742440	-2253.7052424
$CuCl(H_2O)_2^0$ C_2 #1	-2253.3579568	-2253.4796449	-2253.7091056
$CuCl(H_2O)_2^0$ C_s #2	-2253.3554811	-2253.4769169	-2253.7059544
$CuCl(H_2O)_2^0$ C_s #3	[2+1]	[2+1]	[2+1]
$CuCl(H_2O)_2^0$ C_s #4 [2+1]	-2253.3661287	-2253.4910094	-2253.7220112
$CuCl(H_2O)_2^0$ C_s #5 [2+1]	-2253.3673639	-2253.4894532	-2253.7208973
$CuCl(H_2O)_2^0$ C_s #6	-2253.3603419	-2253.4803688	-2253.7089568
$CuCl(H_2O)_2^0$ C_1 #1 [2+1]	**-2253.3724148**	**-2253.4922607**	**-2253.7224255**
$CuCl(H_2O)_2^0$ C_1 #2 [2+1]	C_1 #1	**-2253.4922884**	**-2253.7224091**
$CuCl(H_2O)_3^0$ C_{3v} #1	-2329.7832224	-2329.9025276	-2330.1574308
$CuCl(H_2O)_3^0$ C_{3v} #2	-2329.7747717	-2329.9056297	-2330.1560999
$CuCl(H_2O)_3^0$ C_3 #2	-2329.7905185	-2329.9111012	**-2330.1631411**
$CuCl(H_2O)_3^0$ C_s #1	-2329.7931906	-2329.9133361 [2+2]	-2330.1601575
$CuCl(H_2O)_3^0$ C_s #2 [2+2]	-2329.7923862 [4+0]	**-2329.9316050**	**-2330.1845926**
$CuCl(H_2O)_3^0$ C_1 #3 [2+2]	**-2329.8137468**	**-2329.9421136**	**-2330.1949447**

	B3LYP/6-31G*	B3LYP/6-31+G*	B3LYP/6-311+G*
$CuCl(H_2O)_4^0$ *C_{4v}* #1	-2406.2026877	-2406.3286656	-2406.6050204
$CuCl(H_2O)_4^0$ *C_{4v}* #2	-2406.1829445	-2406.3255274	-2406.5973612
$CuCl(H_2O)_4^0$ *C_4* #1	-2406.2169790	-2406.3454543	-2406.6190384
$CuCl(H_2O)_4^0$ *C_4* #2	-2406.2150036	*C_4* #1	*C_4* #1
$CuCl(H_2O)_4^0$ *C_{2v}* #1	-2406.2072457	-2406.3356918	-2406.6090353
$CuCl(H_2O)_4^0$ *C_{2v}* #2	-2406.2057332	-2406.3395832	-2406.6142362
$CuCl(H_2O)_4^0$ *C_{2v}* #3	-2406.1890641	*C_{2v}* #1	*C_{2v}* #1
$CuCl(H_2O)_4^0$ *C_{2v}* #4	-2406.2090614	-2406.3419126	-2406.6143234
$CuCl(H_2O)_4^0$ *C_{2v}* #5	-2406.2107695	-2406.3475259	-2406.6199181
$CuCl(H_2O)_4^0$ *C_2* #1	*C_2* #2	*C_2* #2	*C_2* #2
$CuCl(H_2O)_4^0$ *C_2* #2 [3+2]	**-2406.2448739**	**-2406.3748485**	**-2406.6510524**
$CuCl(H_2O)_4^0$ *C_2* #3 [3+2]	-2406.2281482	-2406.3649240	
$CuCl(H_2O)_4^0$ *C_2* #4 [3+2]	**-2406.2426216**	**-2406.3727185**	*C_2* #2?
$CuCl(H_2O)_4^0$ *C_s* #1 [4+1]	-2406.2282862	*C_s* #2	
$CuCl(H_2O)_4^0$ *C_s* #2 [2+3]	-2406.2274059	-2406.3648468	-2406.6407655
$CuCl(H_2O)_4^0$ *C_s* #3 [4+1]	-2406.2113067	-2406.3436044	-2406.6169170
$CuCl(H_2O)_4^0$ *C_s* #4 [3+2]	-2406.2347759	-2406.3636477	-2406.6385051
$CuCl(H_2O)_4^0$ *C_s* #5 [3+2]	**-2406.2452097**	**-2406.3743491**	**-2406.6506638**
$CuCl(H_2O)_4^0$ *C_s* #6 [5+0]	*C_s* #4	*C_s* #4	*C_s* #4
$CuCl(H_2O)_4^0$ *C_s* #7 [2+3]	**-2406.2488355**	**-2406.3807420**	**-2406.6554485**
$CuCl(H_2O)_4^0$ *C_1* #3 [3+2]	**-2406.2413958**	*C_1* #4	*C_1* #4
$CuCl(H_2O)_4^0$ *C_1* #4 [2+2+1]	**-2406.2416373**	**-2406.3850047**	**-2406.6617852**
$CuCl(H_2O)_4^0$ *C_1* #5 [2+3]	**-2406.2451000**	*C_1* #4	*C_1* #4
$CuCl(H_2O)_4^0$ *C_1* #6 [4+1]	*C_1* #7		
$CuCl(H_2O)_4^0$ *C_1* #7 [3+1+Cl^-]	**-2406.2388846**	**-2406.3584957**	**-2406.6343183**
$CuCl(H_2O)_4^0$ *C_1* #8 [3+2]	*C_1* #7	**-2406.3721950**	**-2406.6470431**
$CuCl(H_2O)_4^0$ *C_1* #9 [4+1]	**-2406.2339589**	**-2406.3593708**	**-2406.6342841**
$CuCl(H_2O)_4^0$ *C_1* #10 [3+2]		**-2406.3754192**	**-2406.6506988**
$CuCl(H_2O)_4^0$ *C_1* #11 [3+2]	*C_1* #12	**-2406.3656452**	**-2406.6409264**
$CuCl(H_2O)_4^0$ *C_1* #12 [2+2+Cl^-]	**-2406.2402657**	*C_1* #11	*C_1* #11
$CuCl(H_2O)_5^0$ [5+Cl^-] *C_{2v}* #1	-2482.6386380	-2482.7747505	-2483.0681871
$CuCl(H_2O)_5^0$ [5+Cl^-] *C_{2v}* #2	-2482.6367592	-2482.7695283	-2483.0659104
$CuCl(H_2O)_5^0$ [2+4] *C_{2v}* #3	[5+Cl^-] -2482.6347348	-2482.7901337	-2483.0879301
$CuCl(H_2O)_5^0$ [2+4] *C_{2v}* #4	-2482.6449724	-2482.7907959	-2483.0882331
$CuCl(H_2O)_5^0$ [4+2] *C_{2v}* #5	[5+Cl^-] -2482.6494813	-2482.7754100	-2483.0697831
$CuCl(H_2O)_5^0$ [2+4] *C_{2v}* #6	[3+2+Cl^-] -2482.6556715	-2482.7939585	-2483.0909536
$CuCl(H_2O)_5^0$ [2+4] *C_{2v}* #1	-2482.6669526	-2482.8138047	-2483.1141686
$CuCl(H_2O)_5^0$ [2+4] *C_2* #1	-2482.6777075	-2482.8180417	-2483.1173147
$CuCl(H_2O)_5^0$ [2+4] *C_s* #1	**-2482.6827541**	**-2482.8241797**	**-2483.1222281**
$CuCl(H_2O)_7^0$ [2+4] *C_{2v}* #1	-2635.5262693	-2635.6975786	-2636.0460061
$CuCl(H_2O)_7^0$ [2+4] *C_2* #1	-2635.5432811	-2635.7061480	-2636.0528462
$CuCl(H_2O)_7^0$ [2+4] *C_s* #1	**-2635.5473447**	**-2635.7089033**	**-2636.0547098**
Cu_3Cl_3 D_{3h}	**-6301.6408218**	**-6301.9215215**	**-6302.4723577**

	B3LYP/6-31G*	B3LYP/6-31+G*	B3LYP/6-311+G*
$CuCl_2^-$ $D_{\infty h}$	**-2560.8349190**	**-2560.9665174**	**-2561.1785517**
$CuCl_2(H_2O)^-$ C_{2v} #1	-2637.2480540	-2637.3823925	-2637.6168927
$CuCl_2(H_2O)^-$ C_{2v} #2	-2637.2409652	water dissociates	water dissociates
$CuCl_2(H_2O)^-$ C_{2v} #3 [2+1]	-2637.2671486	-2637.4041211	-2637.6376697
$CuCl_2(H_2O)^-$ C_s #1	C_{2v} #3 [2+1]	C_{2v} #3 [2+1]	C_{2v} #3 [2+1]
$CuCl_2(H_2O)^-$ C_s #2	C_s #3 [2+1]	C_s #3 [2+1]	C_s #3 [2+1]
$CuCl_2(H_2O)^-$ C_s #3 [2+1]	**-2637.2601532**	**-2637.4042699**	**-2637.6380211**
$CuCl_2(H_2O)^-$ C_s #4 [2+1]	-2637.2588475	-2637.4028044	-2637.6366751
$CuCl_2(H_2O)_2^-$ D_{2h} #1	-2713.6510768	-2713.7967225	-2714.0534052
$CuCl_2(H_2O)_2^-$ D_{2h} #2	-2713.6364566	water dissociates	water dissociates
$CuCl_2(H_2O)_2^-$ D_{2h} #3 [2+2]	-2713.6931560	-2713.8396760	-2714.0946584
$CuCl_2(H_2O)_2^-$ C_{2h} #1	D_{2h} #3 [2+2]	D_{2h} #3 [2+2]	D_{2h} #3 [2+2]
$CuCl_2(H_2O)_2^-$ C_{2h} #2	C_{2h} #3 [2+2]	C_{2h} #3 [2+2]	C_{2h} #3 [2+2]
$CuCl_2(H_2O)_2^-$ C_{2h} #3 [2+2]	-2713.6842637	**-2713.8409063**	**-2714.0962312**
$CuCl_2(H_2O)_2^-$ C_{2v} #1	water dissociates	water dissociates	water dissociates
$CuCl_2(H_2O)_2^-$ C_{2v} #2	water dissociates	water dissociates	water dissociates
$CuCl_2(H_2O)_2^-$ C_{2v} #3 [2+2]	-2713.6841380	-2713.8401278	-2714.0955357
$CuCl_2(H_2O)_2^-$ C_{2v} #4 [2+2]	**-2713.6835944**	-2713.8401493	**-2714.0956008**
$CuCl_2(H_2O)_2^-$ C_{2v} #5 [2+2]	-2713.6985661	-2713.8400971	-2714.0951286
$CuCl_2(H_2O)_2^-$ C_2 #1 [2+2]	-2713.6844636	n/a	n/a
$CuCl_2(H_2O)_2^-$ C_2 #2 [2+2]	**-2713.6845618**	C_2 #4 [2+2]	C_2 #4 [2+2]
$CuCl_2(H_2O)_2^-$ C_2 #3 [2+2]	n/a	n/a	n/a
$CuCl_2(H_2O)_2^-$ C_2 #4 [2+2]	**-2713.7002491**	**-2713.8408848**	**-2714.0961955**
$CuCl_2(H_2O)_2^-$ C_i #1 [2+2]	-2713.6843203	n/a	n/a
$CuCl_2(H_2O)_2^-$ C_s #1 [2+2]	**-2713.7002018**	-2713.8403134	**-2714.0955850**
$CuCl_2(H_2O)_2^-$ C_s #2 [2+2]	-2713.6842576	n/a	n/a
$CuCl_2(H_2O)_2^-$ C_s #3 [2+2]	n/a	**-2713.8403139**	n/a
$CuCl_2(H_2O)_2^-$ C_s #4 [2+2]	n/a	C_{2v} #4 [2+2]	n/a
$CuCl_2(H_2O)_2^-$ C_1 #1 [2+2]	**-2713.7004612**	**-2713.8451575**	**-2714.1011459**

	B3LYP/6-31G*	B3LYP/6-31+G*	B3LYP/6-311+G*
$CuCl_2(H_2O)_3^-$ D_{3h} #1	-2790.0659066	-2790.2117949	-2790.4924505
$CuCl_2(H_2O)_3^-$ D_{3h} #2	-2790.0410221	water dissociates	water dissociates
$CuCl_2(H_2O)_3^-$ D_{3h} #3 [2+3]	-2790.1279376	-2790.2742607	-2790.5505954
$CuCl_2(H_2O)_3^-$ C_{3h} #1	D_{3h} #3 [2+3]	D_{3h} #3 [2+3]	D_{3h} #3 [2+3]
$CuCl_2(H_2O)_3^-$ C_{3v} #1 [4+Cl^-]	-2790.1076877	-2790.2474342	-2790.5293521
$CuCl_2(H_2O)_3^-$ C_{3v} #2 [2+3]	-2790.1282316	-2790.2746893	-2790.5515305
$CuCl_2(H_2O)_3^-$ C_{2v} #1 [3+2]	-2790.1157452	-2790.2604080	-2790.5385952
$CuCl_2(H_2O)_3^-$ C_{2v} #2 [3+2 Cl^-]	-2790.0908986	D_{3h} #3 [2+3]	D_{3h} #3 [2+3]
$CuCl_2(H_2O)_3^-$ C_{2v} #3 [2+3]	n/a	n/a	n/a
$CuCl_2(H_2O)_3^-$ C_{2v} #4 [2+3]	-2790.1184423	-2790.2742646	-2790.5506544
$CuCl_2(H_2O)_3^-$ C_3 #1 [4+Cl^-]	-2790.1173951	-2790.2591914	-2790.5381920
$CuCl_2(H_2O)_3^-$ C_3 #2 [2+3]	D_{3h} #3 [2+3]	C_{3v} #2 [2+3]	C_{3v} #2 [2+3]
$CuCl_2(H_2O)_3^-$ C_2 #1 [3+2 Cl^-]	-2790.1244023	-2790.2478565	-2790.5307406
$CuCl_2(H_2O)_3^-$ C_2 #2 [3+2]	-2790.1230816	-2790.2736754	-2790.5534216
$CuCl_2(H_2O)_3^-$ C_2 #3 [3+2]	-2790.1080749	-2790.2621407	-2790.5403735
$CuCl_2(H_2O)_3^-$ C_2 #4 [2+2+1]		**-2790.2849410**	
$CuCl_2(H_2O)_3^-$ C_2 #5 [2+3]	**-2790.1279621**	**-2790.2813590**	-2790.5592316
$CuCl_2(H_2O)_3^-$ C_2 #6 [2+3]	C_2 #5 [2+3]	C_2 #5 [2+3]	C_2 #5 [2+3]
$CuCl_2(H_2O)_3^-$ C_2 #7 [2+3]	**-2790.1288266**	C_2 #5 [2+3]	C_2 #5 [2+3]
$CuCl_2(H_2O)_3^-$ C_2 #8 [2+3]	n/a	n/a	n/a
$CuCl_2(H_2O)_3^-$ C_s #1 [4+ Cl^-]	C_s #2 [3+ 1+Cl^-]	C_s #2 [3+ 1+Cl^-]	C_s #2 [3+ 1+Cl^-]
$CuCl_2(H_2O)_3^-$ C_s #2 [3+ 1+Cl^-]	-2790.1244046	-2790.2644995	-2790.5447467
$CuCl_2(H_2O)_3^-$ C_s #3 [2+3]	-2790.1064421	-2790.2750180	-2790.5522851
$CuCl_2(H_2O)_3^-$ C_s #4 [2+3]	-2790.1287026	**-2790.2757139**	**-2790.5528997**
$CuCl_2(H_2O)_3^-$ C_s #5 [2+3]	C_s #4 [2+3]	-2790.2746511	-2790.5515315
$CuCl_2(H_2O)_3^-$ C_s #6 [2+3]	n/a	n/a	n/a
$CuCl_2(H_2O)_3^-$ C_s #7 [2+3]	n/a	n/a	n/a
$CuCl_2(H_2O)_3^-$ C_s #8 [2+3]	n/a	n/a	n/a
$CuCl_2(H_2O)_3^-$ C_s #9 [2+3]	n/a	n/a	n/a
$CuCl_2(H_2O)_3^-$ C_1 #1 [4+ Cl^-]	n/a	n/a	n/a
$CuCl_2(H_2O)_3^-$ C_1 #2 [3+1+Cl^-]	**-2790.1308106**	C_1 #3 [2+2+Cl^-]	C_1 #3 [2+2+Cl^-]
$CuCl_2(H_2O)_3^-$ C_1 #3 [2+2+Cl^-]	n/a	**-2790.2718250**	
$CuCl_2(H_2O)_3^-$ C_1 #4 [3+2Cl^-]	**-2790.1252609**	**-2790.2500574**	**-2790.5317624**
$CuCl_2(H_2O)_3^-$ C_1 #5 [3+2]	**-2790.1243157**	C_2 #4 [2+2+1]	**-2790.5553606**
$CuCl_2(H_2O)_3^-$ C_1 #6 [3+2]	**-2790.1309212**		
$CuCl_2(H_2O)_3^-$ C_1 #7 [3+2]	**-2790.1322654**	**-2790.2782435**	

	B3LYP/6-31G*	B3LYP/6-31+G*	B3LYP/6-311+G*
$CuCl_2(H_2O)_4^-$ D_{4h} #2 [2+4]	-2866.5412721	-2866.7065301	-2867.0047183
$CuCl_2(H_2O)_6^-$ D_{3d} #1 [2+6]	-3019.3672587	-3019.5734901	-3019.9155143
$CuCl_2(H_2O)_6^-$ S_6 #1 [2+6]	**-3019.4237492**	**-3019.6022070**	**-3019.9470318**
$CuCl_2(H_2O)_6^-$ S_6 #2 [2+6]	-3019.4241962	**-3019.5975650**	**-3019.9412205**
$CuCl_2(H_2O)_6^-$ D_3 #1 [2+6]	-3019.3967212<br>-3019.3957132	-3019.5853292	-3019.9278841
$CuCl_2(H_2O)_6^-$ D_3 #2 [2+6]	**-3019.4237628**	**-3019.6022206**	**-3019.9470427**
$CuCl_2(H_2O)_6^-$ C_{3h} #1 [2+6]	**-3019.4237569**	**-3019.6022142**	**-3019.9470382**
$CuCl_2(H_2O)_6^-$ C_3 #1 [2+6]	**-3019.4103347**	**-3019.5975454**	

	B3LYP/6-31G*	B3LYP/6-31+G*	B3LYP/6-311+G*
$CuCl_3^{2-}$ D_{3h}	**-3020.9853719**	**-3021.1386970**	**-3021.3795724**
$CuCl_3(H_2O)^{2-}$ C_{2v} #1 [3+1]	**-3097.4305727**	**-3097.5946536**	**-3097.8576635**
$CuCl_3(H_2O)^{2-}$ C_s #1	[2+1+Cl^-]	C_s #1 [3+1]	C_s #1 [3+1]
$CuCl_3(H_2O)^{2-}$ C_s #2	[2+2Cl^-]	C_{2v} #1 [3+1]	[2+2Cl^-]
$CuCl_3(H_2O)^{2-}$ C_s #1 [3+1]	[2+1+Cl^-]	-3097.5873280	-3097.8502945
$CuCl_3(H_2O)_2^{2-}$ C_{2v} #1 [3+2]	-3173.8714041	**-3174.0449702**	**-3174.3303152**
$CuCl_3(H_2O)_2^{2-}$ C_{2v} #2 [3+2]	**-3173.8733176**	**-3174.0482626**	**-3174.3335083**
$CuCl_3(H_2O)_2^{2-}$ C_s #1 [3+2]	**-3173.8714921**	n/a	n/a
$CuCl_3(H_2O)_3^{2-}$ D_{3h} [3+3]	**-3250.3111992**	**-3250.4994569**	**-3250.8069252**
$CuCl_3(H_2O)_3^{2-}$ C_s #1 [3+3]	**-3250.3121977**	**-3250.4967064**	**-3250.8042019**
$CuCl_3(H_2O)_4^{2-}$ C_{2v} #1 [3+4]	**-3326.7474123**	**-3326.9458592**	**-3327.2754722**
$CuCl_3(H_2O)_4^{2-}$ C_{2v} #2 [3+4]	**-3326.7489186**	**-3326.9427571**	**-3327.2723895**
$CuCl_3(H_2O)_5^{2-}$ C_{2v} #1 [3+5]	-3403.1822985	**-3403.3897103**	**-3403.7414347**
$CuCl_3(H_2O)_5^{2-}$ C_s #1 [3+5]	-3403.1865938	n/a	n/a
$CuCl_3(H_2O)_5^{2-}$ C_s #2 [3+5]	-3403.1834443	n/a	n/a
$CuCl_3(H_2O)_6^{2-}$ D_{3h} #1 [3+6]	**-3479.6134329**	**-3479.8313163**	**-3480.2050082**
$CuCl_4^{3-}$ T_d	-3481.0093591	-3481.2035710	-3481.4726426
$CuCl_4(H_2O)_6^{3-}$ T_d [4+6]	-3939.7106421	**-3939.9626973**	**-3940.3668069**

	MP2/6-31G*	MP2/6-31+G*	MP2/6-311+G*
Cu^+ K_h	**-1638.5962821** **-1638.2003534**	**-1638.7476775** **-1638.2293274**	**-1638.9805098**
H_2O C_{2v}	**-76.1968478**	**-76.2097766**	**-76.2447096**
Cl^- K_h	**-459.6521044**	**-459.6711454**	**-459.7035702**
$CuCl^0$ $C_{\infty v}$ (CC)	**-2098.1032911**	**-2098.1535167**	
$CuCl^0$ $C_{\infty v}$	**-2098.5540609**	**-2098.7085152**	**-2098.9680301**
$CuCl(H_2O)_1^0$ C_{2v}	-2174.8284281	-2174.9691928	-2175.2657729
$CuCl(H_2O)_1^0$ C_s #3	**-2174.8294309**	**-2174.9703640**	**-2175.2660705**
$CuCl(H_2O)_1^0$ C_1 #1 trip (DM)	**-2174.7421607**	**-2174.8223823**	
$CuCl(H_2O)_2^0$ C_{2v} #3	-2251.0366877	-2251.1868665	-2251.5175972
$CuCl(H_2O)_2^0$ C_{2v} #4	-2251.0372350	-2251.1839158	-2251.5164746
$CuCl(H_2O)_2^0$ C_2 #1	-2251.0408622	-2251.1899787	-2251.5212711
$CuCl(H_2O)_2^0$ C_s #1 (DM)	**-2251.0423651**	*C_{2v} #3*	
$CuCl(H_2O)_2^0$ C_s #1 trip (DM)	**-2250.9650198**	**-2251.0497549**	
$CuCl(H_2O)_2^0$ C_s #2	-2251.0373061	*C_{2v} #3*	-2251.5177204
$CuCl(H_2O)_2^0$ C_s #3	[2+1]	[2+1]	[2+1]
$CuCl(H_2O)_2^0$ C_s #4	-2251.0507942	-2251.2013554	-2251.5344791
$CuCl(H_2O)_2^0$ C_s #5	-2251.0515393	-2251.1998461	-2251.5331288
$CuCl(H_2O)_2^0$ C_s #6	-2251.0434005	-2251.1903309	-2251.5206648
$CuCl(H_2O)_2^0$ [2+1] C_1 #1	**-2251.0519521**	**-2251.2027781**	**-2251.5348470**
$CuCl(H_2O)_2^0$ [2+1] C_1 #2	**-2251.0548024**	C_1 #1	C_1 #1
$CuCl(H_2O)_3^0$ C_{3v} #1	-2327.2532032	-2327.4019525	-2327.7736628
$CuCl(H_2O)_3^0$ C_{3v} #2	-2327.2443184	-2327.4045743	-2327.7701827
$CuCl(H_2O)_3^0$ C_3 #1 (CC)	(Cl^-) **-2326.7851330**	(Cl^-) **-2326.8507383**	
$CuCl(H_2O)_3^0$ C_3 #1 (DM)	C_1 #2	C_1 #2	
$CuCl(H_2O)_3^0$ C_3 #1 trip (DM)	**-2327.1904907**	**-2327.2720786**	
$CuCl(H_2O)_3^0$ C_3 #2	-2327.2591302	-2327.4097750	-2327.7780949
$CuCl(H_2O)_3^0$ C_s #1	-2327.2633330	-2327.4207765 [3+1]	-2327.7756365
$CuCl(H_2O)_3^0$ C_s #2 [2+2]	-2327.2711128	**-2327.4316545**	**-2327.7997868**
$CuCl(H_2O)_3^0$ [2+2] C_1 #1	**-2327.2835065**	**-2327.4417923**	**-2327.8096380**
$CuCl(H_2O)_3^0$ [3+1] C_1 #2 (DM)	**-2327.2730914**	**-2327.4256450**	

	MP2/6-31G*	MP2/6-31+G*	MP2/6-311+G*
$CuCl(H_2O)_4^0$ C_{4v} #1	-2403.4614290	-2403.6182943	-2404.0257195
$CuCl(H_2O)_4^0$ C_{4v} #2	-2403.4409611	-2403.6143402	-2404.0150908
$CuCl(H_2O)_4^0$ C_4 #1	-2403.4758379	-2403.6426156	-2404.0453588
$CuCl(H_2O)_4^0$ C_4 #2	-2403.4722181	C_4 #1	-2404.0381064
$CuCl(H_2O)_4^0$ C_{2v} #1	-2403.4662394	-2403.6241005	-2404.0282057
$CuCl(H_2O)_4^0$ C_{2v} #2	-2403.4652629	-2403.6279691	-2404.0307213
$CuCl(H_2O)_4^0$ C_{2v} #3	C_{2v} #1	C_{2v} #1	C_{2v} #1
$CuCl(H_2O)_4^0$ C_{2v} #4	-2403.4656167	-2403.6315869	-2404.0325172
$CuCl(H_2O)_4^0$ C_{2v} #5	-2403.4695860	-2403.6378458	-2404.0388679
$CuCl(H_2O)_4^0$ C_2 #1	C_2 #2	C_2 #3	C_2 #3
$CuCl(H_2O)_4^0$ [3+2] C_2 #2	**-2403.5011722**	**-2403.6631645**	-2404.0673497
$CuCl(H_2O)_4^0$ [3+2] trip C_2 #2 (DM)	-2403.4041286	-2403.5106992	
$CuCl(H_2O)_4^0$ [3+2] C_2 #3	-2403.4849775	-2403.6542035	-2404.0579108
$CuCl(H_2O)_4^0$ [3+2] C_2 #4	**-2403.4995132**	C_2 #2?	-2404.0655275
$CuCl(H_2O)_4^0$ [4+1] C_s #1	-2403.4849044	C_s #2	-2404.0519260
$CuCl(H_2O)_4^0$ [2+3] C_s #2	-2403.4874035	-2403.6560040	-2404.0604447
$CuCl(H_2O)_4^0$ [4+1] C_s #3	-2403.4690714	-2403.6334782	-2404.0349514
$CuCl(H_2O)_4^0$ [3+2] C_s #4	-2403.4908638	-2403.6530330	-2404.0560063
$CuCl(H_2O)_4^0$ [3+2] C_s #5	**-2403.5020060**	**-2403.6621899**	**-2404.0671285**
$CuCl(H_2O)_4^0$ [3+2] C_s #6	C_s #4	C_s #4	C_s #4
$CuCl(H_2O)_4^0$ [2+3] C_s #7	**-2403.5057677**	**-2403.6710515**	**-2404.0734989**
$CuCl(H_2O)_4^0$ C_1 #1 [3+2]	n/a	n/a	**-2404.0673559**
$CuCl(H_2O)_4^0$ C_1 #2 [3+2]	n/a	n/a	**-2404.0666442**
$CuCl(H_2O)_4^0$ C_1 #3 [3+2]	**-2403.4996199**	C_1 #4	C_1 #4
$CuCl(H_2O)_4^0$ C_1 #4 [2+2+1]	**-2403.5024726**	**-2403.6730331**	**-2404.0783217**
$CuCl(H_2O)_4^0$ C_1 #5 [2+3]	**-2403.5014285**	C_1 #4	C_1 #4
$CuCl(H_2O)_4^0$ C_1 #6 [4+1]	C_1 #7	**-2403.6494174**	C_1 #8
$CuCl(H_2O)_4^0$ C_1 #7 [3+1+Cl^-]	**-2403.4972366**	C_1 #6	**-2404.0484244**
$CuCl(H_2O)_4^0$ C_1 #8 [3+2]	**-2403.4977914**	**-2403.6630587**	**-2404.0662202**
$CuCl(H_2O)_4^0$ C_1 #9 [4+1]	C_1 #7	**-2403.6483487**	**-2404.0524853**
$CuCl(H_2O)_4^0$ C_1 #10 [3+2]	**-2403.5031311**	**-2403.6693431**	**-2404.0678898**
$CuCl(H_2O)_4^0$ C_1 #11 [3+2]	C_1 #12	**-2403.6538836**	**-2404.0579364**
$CuCl(H_2O)_4^0$ C_1 #12 [2+2+Cl^-]	**-2403.4968260** **-2403.4957271**		

	MP2/6-31G*	MP2/6-31+G*	MP2/6-311+G*
$CuCl(H_2O)_5^0$ [2+4] C_{2v} #1	[5+Cl^-] -2479.6875315	-2479.8533809	-2480.2907637
$CuCl(H_2O)_5^0$ [2+4] C_{2v} #2	[5+Cl^-] -2479.6854063	-2479.8514351	-2480.2885406
$CuCl(H_2O)_5^0$ [2+4] C_{2v} #3	[5+Cl^-] -2479.6817751	-2479.8678278	-2480.3070075
$CuCl(H_2O)_5^0$ [2+4] C_{2v} #4	-2479.6900273	-2479.8684287	-2480.3081227
$CuCl(H_2O)_5^0$ [4+2] C_{2v} #5	[5+Cl^-] -2479.6937073	-2479.8542876	-2480.2901242
$CuCl(H_2O)_5^0$ [2+4] C_{2v} #6	[5+Cl^-] -2479.6959334	-2479.8717438	-2480.3106133
$CuCl(H_2O)_5^0$ [3+3] trip C_s #1 (CC)		**-2479.7465771**	
$CuCl(H_2O)_5^0$ [3+2+Cl^-] C_1 #1 (CC)	**-2479.7224811**		
$CuCl(H_2O)_5^0$ [3+3] C_1 #2 (CC)		**-2479.8907528**	
$CuCl(H_2O)_5^0$ [2+4] C_{2v} #1	-2479.7141082	-2479.8931521	-2480.3351615
$CuCl(H_2O)_5^0$ [2+4] C_2 #1	-2479.7219238	-2479.8973124	-2480.3389407
$CuCl(H_2O)_5^0$ [2+4] C_s #1	**-2479.7264615**	**-2479.9034214**	**-2480.3430768**
$CuCl(H_2O)_7^0$ [2+4] C_{2v} #1	-2632.1484648	-2632.3538257	-2632.8697078
$CuCl(H_2O)_7^0$ [2+4] C_2 #1	-2632.1618697	-2632.3627109	-2632.8775972
$CuCl(H_2O)_7^0$ [2+4] C_s #1	**-2632.1645243**	**-2632.3652165**	**-2632.8795372**
Cu_3Cl_3 D_{3h}	**-6295.9736016**	**-6296.3419144**	**-6297.1085158**

	MP2/6-31G*	MP2/6-31+G*	MP2/6-311+G*
$CuCl_2^-$ $D_{\infty h}$	**-2558.3450958**	**-2558.5044656**	**-2558.7925470**
$CuCl_2(H_2O)^-$ C_{2v} #1	-2634.5430617	-2634.7091780	-2635.0335296
$CuCl_2(H_2O)^-$ C_{2v} #2	-2634.5350601	water dissociates	-2635.0271715
$CuCl_2(H_2O)^-$ C_{2v} #3 [2+1]	**-2634.5640843**	-2634.7313586	-2635.0546132
$CuCl_2(H_2O)^-$ C_s #1	C_{2v} #3 [2+1]	C_{2v} #3 [2+1]	C_{2v} #3 [2+1]
$CuCl_2(H_2O)^-$ C_s #2	C_s #3 [2+1]	C_s #3 [2+1]	C_s #3 [2+1]
$CuCl_2(H_2O)^-$ C_s #3 [2+1]	**-2634.5645418**	**-2634.7316428**	C_s #4 [2+1]
$CuCl_2(H_2O)^-$ C_s #4 [2+1]	-2634.5576430	-2634.7291601	-2635.0522213
$CuCl_2(H_2O)_2^-$ D_{2h} #1	-2710.7290948	-2710.9119714	-2711.2724071
$CuCl_2(H_2O)_2^-$ D_{2h} #2	water dissociates	water dissociates	water dissociates
$CuCl_2(H_2O)_2^-$ D_{2h} #3 [2+2]	-2710.7771850	-2710.9560358	-2711.3143510
$CuCl_2(H_2O)_2^-$ C_{2h} #1	D_{2h} #3 [2+2]	D_{2h} #3 [2+2]	D_{2h} #3 [2+2]
$CuCl_2(H_2O)_2^-$ C_{2h} #2	C_{2h} #3 [2+2]	C_{2h} #3 [2+2]	C_{2h} #3 [2+2]
$CuCl_2(H_2O)_2^-$ C_{2h} #3 [2+2]	-2710.7717045	-2710.9575225	-2711.3149478
$CuCl_2(H_2O)_2^-$ C_{2v} #1	water dissociates	water dissociates	water dissociates
$CuCl_2(H_2O)_2^-$ C_{2v} #2	water dissociates	water dissociates	water dissociates
$CuCl_2(H_2O)_2^-$ C_{2v} #3 [2+2]	-2710.7716018	-2710.9556938	-2711.3136351
$CuCl_2(H_2O)_2^-$ C_{2v} #4 [2+2]	**-2710.7775934**	-2710.9568819	D_{2h} #3 [2+2]
$CuCl_2(H_2O)_2^-$ C_{2v} #5 [2+2]	-2710.7825235	-2710.9567250	**-2711.3149145**
$CuCl_2(H_2O)_2^-$ C_2 #1 [2+2]	**-2710.7719316**	-2710.9605715	**-2711.3149648**
$CuCl_2(H_2O)_2^-$ C_2 #2 [2+2]	C_2 #1 [2+2]	C_2 #4 [2+2]	-2711.3183599
$CuCl_2(H_2O)_2^-$ C_2 #3 [2+2]	n/a	n/a	D_{2h} #3 [2+2]
$CuCl_2(H_2O)_2^-$ C_2 #4 [2+2]	**-2710.7834349**	**-2710.9575929**	n/a
$CuCl_2(H_2O)_2^-$ C_i #1 [2+2]	n/a	n/a	n/a
$CuCl_2(H_2O)_2^-$ C_s #1 [2+2]	**-2710.7833611**	**-2710.9571511**	n/a
$CuCl_2(H_2O)_2^-$ C_s #2 [2+2]	-2710.7716053	n/a	n/a
$CuCl_2(H_2O)_2^-$ C_s #3 [2+2]	C_s #1 [2+2]	C_s #1 [2+2]	C_{2v} #5 [2+2]
$CuCl_2(H_2O)_2^-$ C_s #4 [2+2]	C_{2v} #4 [2+2]	n/a	C_{2h} #3 [2+2]
$CuCl_2(H_2O)_2^-$ C_1 #1 [2+2]	**-2710.7845006**	**-2710.9622667**	**-2711.3205823**

	MP2/6-31G*	MP2/6-31+G*	MP2/6-311+G*
$CuCl_2(H_2O)_3^-$ D_{3h} #1	-2786.9296909	-2787.1155897	-2787.5146915
$CuCl_2(H_2O)_3^-$ D_{3h} #2	-2786.9013113	water dissociates	water dissociates
$CuCl_2(H_2O)_3^-$ D_{3h} #3 [2+3]	-2786.9987588	-2787.1800530	-2787.5731704
$CuCl_2(H_2O)_3^-$ C_{3h} #1	D_{3h} #3 [2+3]	D_{3h} #3 [2+3]	D_{3h} #3 [2+3]
$CuCl_2(H_2O)_3^-$ C_{3v} #1 [4+Cl^-]	-2786.9789228	-2787.1491888	-2787.5512955
$CuCl_2(H_2O)_3^-$ C_{3v} #2 [2+3]	D_{3h} #3 [2+3]	-2787.1809868	D_{3h} #3 [2+3]
$CuCl_2(H_2O)_3^-$ C_{2v} #1 [3+2]	-2786.9857277	-2787.1672042	-2787.5625190
$CuCl_2(H_2O)_3^-$ C_{2v} #2 [3+2 Cl^-]	-2786.9591496	C_{2v} #3 [2+3]	C_{2v} #3 [2+3]
$CuCl_2(H_2O)_3^-$ C_{2v} #3 [2+3]	n/a	-2787.1801205	-2787.5733908
$CuCl_2(H_2O)_3^-$ C_{2v} #4 [2+3]	-2786.9909281	-2787.1802448	-2787.5734324
$CuCl_2(H_2O)_3^-$ C_3 #1 [4+Cl^-]	-2786.9859012	-2787.1601522	**-2787.5576780**
$CuCl_2(H_2O)_3^-$ C_3 #2 [2+3]	D_{3h} #3 [2+3]	C_{3v} #2 [2+3]	**-2787.5729143**
$CuCl_2(H_2O)_3^-$ C_2 #1 [3+2 Cl^-]	-2786.9935298	-2787.1419886	-2787.5450159
$CuCl_2(H_2O)_3^-$ C_2 #2 [3+2]	-2786.9929270	-2787.1791281	-2787.5750023
$CuCl_2(H_2O)_3^-$ C_2 #3 [4+2]	-2786.9801443	-2787.1684045	-2787.5625653
$CuCl_2(H_2O)_3^-$ C_2 #4 [2+2+1]		**-2787.1914056**	
$CuCl_2(H_2O)_3^-$ C_2 #5 [2+3]	-2787.0008321	-2787.1876386	**-2787.5817319**
$CuCl_2(H_2O)_3^-$ C_2 #6 [2+3]	C_2 #5 [2+3]	C_2 #5 [2+3]	**-2787.5741467**
$CuCl_2(H_2O)_3^-$ C_2 #7 [2+3]	**-2786.9993655**	-2787.1815281	C_2 #6 [2+3]
$CuCl_2(H_2O)_3^-$ C_2 #8 [2+3]	n/a	-2787.1846930	C_2 #6 [2+3]
$CuCl_2(H_2O)_3^-$ C_s #1 [4+ Cl^-]	C_s #2 [3+ 1+Cl^-]	C_s #2 [3+ 1+Cl^-]	-2787.5559202
$CuCl_2(H_2O)_3^-$ C_s #2 [3+ 1+Cl^-]	-2786.9917350	-2787.1640197	-2787.5614199
$CuCl_2(H_2O)_3^-$ C_s #3 [2+3]	-2786.9828099	-2787.1797160	-2787.5722110
$CuCl_2(H_2O)_3^-$ C_s #4 [2+3]	**-2786.9994712**	-2787.1818754	-2787.5741379
$CuCl_2(H_2O)_3^-$ C_s #5 [2+3]	C_s #4 [2+3]	-2787.1809856	-2787.5739440
$CuCl_2(H_2O)_3^-$ C_s #6 [2+3]	n/a	C_{2v} #4 [2+3]	C_{2v} #4 [2+3]
$CuCl_2(H_2O)_3^-$ C_s #7 [2+3]	n/a	C_s #4 [2+3]	C_s #5 [2+3]
$CuCl_2(H_2O)_3^-$ C_s #8 [2+3]	n/a	C_s #5 [2+3]	C_s #5 [2+3]
$CuCl_2(H_2O)_3^-$ C_s #9 [2+3]	n/a	C_{2v} #4 [2+3]	C_{2v} #3 [2+3]
$CuCl_2(H_2O)_3^-$ C_1 #1 [3+2] (CC)	**-2786.9976313**	**-2787.1662017**	
$CuCl_2(H_2O)_3^-$ C_1 #2 [3+2] (CC)	**-2787.0037130**	**-2787.1701309**	
$CuCl_2(H_2O)_3^-$ C_1 #1 [4+ Cl^-]	n/a	n/a	C_1 #2 [3+1+Cl^-]
$CuCl_2(H_2O)_3^-$ C_1 #2 [3+1+Cl^-]	**-2786.9950821**	C_1 #3 [2+2+Cl^-]	**-2787.5670876**
$CuCl_2(H_2O)_3^-$ C_1 #3 [2+2+Cl^-]	n/a	**-2787.1710037**	**-2787.5694389**
$CuCl_2(H_2O)_3^-$ C_1 #4 [3+2Cl^-]	**-2786.9937215**	**-2787.1437275**	**-2787.5453028**
$CuCl_2(H_2O)_3^-$ C_1 #5 [3+2]	**-2786.9936737**	C_2 #4 [2+2+1]	**-2787.5757681**
$CuCl_2(H_2O)_3^-$ C_1 #6 [3+2]	**-2787.0066715**		**-2787.5867622**
$CuCl_2(H_2O)_3^-$ C_1 #7 [3+2]	n/a	n/a	n/a

	MP2/6-31G*	MP2/6-31+G*	MP2/6-311+G*
$CuCl_2(H_2O)_4^-$ D_{4h} #2 [2+4]	-2863.1995788	-2863.4026784	-2863.8309547
$CuCl_2(H_2O)_4^-$ C_2 #1 [4+2Cl^-] (CC)	**-2863.2214003**	**-2863.3833977**	
$CuCl_2(H_2O)_6^-$ D_{3d} #1 [2+6]	-3015.6099585	-3015.8496694	-3016.3451829
$CuCl_2(H_2O)_6^-$ S_6 #1 [2+6]	**-3015.6586496**	**-3015.8717602**	-3016.3718349
$CuCl_2(H_2O)_6^-$ S_6 #2 [2+6]	**-3015.6591437**	**-3015.8781905**	**-3016.3756813**
$CuCl_2(H_2O)_6^-$ D_3 #1 [2+6]	-3015.6349968	-3015.8610674	-3016.3582992
$CuCl_2(H_2O)_6^-$ D_3 #2 [2+6]	**-3015.6586496**	**-3015.8717600**	**-3016.3718348**
$CuCl_2(H_2O)_6^-$ C_{3h} #1 [2+6]	**-3015.6586490**	**-3015.8717562**	-3016.3718287

	MP2/6-31G*	MP2/6-31+G*	MP2/6-311+G*
$CuCl_3^{2-}$ D_{3h}	**-3017.8885044**	**-3018.0740453**	**-3018.3949971**
$CuCl_3(H_2O)^{2-}$ C_{2v} #1 [3+1]	-3094.1234529	**-3094.3208323**	**-3094.6766404**
$CuCl_3(H_2O)^{2-}$ C_s #1	[2+1+Cl^-]	C_s #1 [3+1]	C_s #1 [3+1]
$CuCl_3(H_2O)^{2-}$ C_s #2	[2+2Cl^-]	[2+2Cl^-]	[2+2Cl^-]
$CuCl_3(H_2O)^{2-}$ C_s #1 [3+1]	[2+1+Cl^-]	-3094.3129297	-3094.6687456
$CuCl_3(H_2O)^{2-}$ C_s #3 [3+1]	**-3094.1237845**	n/a	n/a
$CuCl_3(H_2O)_2^{2-}$ C_{2v} #1 [3+2]	-3170.3539475	**-3170.5619277**	**-3170.9527313**
$CuCl_3(H_2O)_2^{2-}$ C_{2v} #2 [3+2]	-3170.3568495	-3170.5651530	-3170.9556116
$CuCl_3(H_2O)_2^{2-}$ C_s #1 [3+2]	[2+2+Cl^-]	n/a	n/a
$CuCl_3(H_2O)_2^{2-}$ C_s #2 [3+2]	**-3170.3568630**	**-3170.5652678**	-3170.9561039
$CuCl_3(H_2O)_3^{2-}$ D_{3h} [3+3]	-3246.5841983	-3246.8070648	-3247.2318806
$CuCl_3(H_2O)_3^{2-}$ C_{3v} [3+3]	**-3246.5843168**	**-3246.8076142**	**-3247.2336520**
$CuCl_3(H_2O)_3^{2-}$ C_1 [3+1+2Cl^-]	**-3246.6057004**	**-3246.7817636**	
$CuCl_3(H_2O)_3^{2-}$ C_s #1 [3+3]	[2+3+Cl^-]	**-3246.8048509**	**-3247.2307957**
$CuCl_3(H_2O)_4^{2-}$ C_{2v} #1 [3+4]	**-3322.8106003**	-3323.0452900	-3323.5061277
$CuCl_3(H_2O)_4^{2-}$ C_{2v} #2 [3+4]	**-3322.8122673**	**-3323.0416051**	**-3323.5019198**
$CuCl_3(H_2O)_4^{2-}$ C_s #1 [3+4]	n/a	**-3323.0452911**	**-3323.5063473**
$CuCl_3(H_2O)_5^{2-}$ C_{2v} #1 [3+5]	-3399.0348265	**-3399.2804767**	**-3399.7763294**
$CuCl_3(H_2O)_5^{2-}$ C_s #1 [3+5]	-3399.0373626	n/a	n/a
$CuCl_3(H_2O)_6^{2-}$ D_{3h} #1 [3+6]	**-3475.2560690**	**-3475.5133247**	-3476.0433679
$CuCl_3(H_2O)_6^{2-}$ C_{3v} #1 [3+6]	n/a	n/a	**-3476.0433709**
$CuCl_4^{3-}$ T_d	-3477.3179496	-3477.5308833	-3477.8850874
$CuCl_4(H_2O)_6^{3-}$ T_d [4+6]	**-3934.7560225**	**-3935.0411390**	**-3935.6065838**
